# Metabolic role of the hepatic valine/3-hydroxyisobutyrate (3-HIB) pathway in fatty liver disease

**DOI:** 10.1016/j.ebiom.2023.104569

**Published:** 2023-04-19

**Authors:** Mona Synnøve Bjune, Laurence Lawrence-Archer, Johnny Laupsa-Borge, Cathrine Horn Sommersten, Adrian McCann, Robert Clay Glastad, Iain George Johnston, Matthias Kern, Matthias Blüher, Gunnar Mellgren, Simon N. Dankel

**Affiliations:** a Mohn Nutrition Research Laboratory, Department of Clinical Science, University of Bergen, Bergen, Norway; b Hormone Laboratory, Haukeland University Hospital, Bergen, Norway; c Mohn Nutrition Research Laboratory, Department of Clinical Medicine, University of Bergen, Bergen, Norway; d Bevital AS, Bergen, Norway; e Department of Mathematics, University of Bergen, Bergen, Norway; f Computational Biology Unit, University of Bergen, Bergen, Norway; g Helmholtz Institute for Metabolic, Obesity and Vascular Research (HI-MAG) of the Helmholtz Zentrum München at the University of Leipzig and University Hospital Leipzig, Leipzig, Germany; h Medical Department III—Endocrinology, Nephrology, Rheumatology, University of Leipzig Medical Center, Leipzig, Germany

**Keywords:** Branched-chain amino acids (BCAA), NAFLD, NASH, Insulin resistance, Lipid metabolism

## Abstract

**Background:**

The valine (branched-chain amino acid) metabolite 3-hydroxyisobutyrate (3-HIB), produced by 3-Hydroxyisobutyryl-CoA Hydrolase (HIBCH), is associated with insulin resistance and type 2 diabetes, but implicated tissues and cellular mechanisms are poorly understood. We hypothesized that HIBCH and 3-HIB regulate hepatic lipid accumulation.

**Methods:**

*HIBCH* mRNA in human liver biopsies (“Liver cohort”) and plasma 3-HIB (“CARBFUNC” cohort) were correlated with fatty liver and metabolic markers. Human Huh7 hepatocytes were supplemented with fatty acids (FAs) to induce lipid accumulation. Following *HIBCH* overexpression, siRNA knockdown, inhibition of PDK4 (a marker of FA β-oxidation) or 3-HIB supplementation, we performed RNA-seq, Western blotting, targeted metabolite analyses and functional assays.

**Findings:**

We identify a regulatory feedback loop between the valine/3-HIB pathway and PDK4 that shapes hepatic FA metabolism and metabolic health and responds to 3-HIB treatment of hepatocytes. *HIBCH* overexpression increased 3-HIB release and FA uptake, while knockdown increased cellular respiration and decreased reactive oxygen species (ROS) associated with metabolic shifts via PDK4 upregulation. Treatment with PDK4 inhibitor lowered 3-HIB release and increased FA uptake, while increasing *HIBCH* mRNA. Implicating this regulatory loop in fatty liver, human cohorts show positive correlations of liver fat with hepatic *HIBCH* and *PDK4* expression (Liver cohort) and plasma 3-HIB (CARBFUNC cohort). Hepatocyte 3-HIB supplementation lowered *HIBCH* expression and FA uptake and increased cellular respiration and ROS.

**Interpretation:**

These data implicate the hepatic valine/3-HIB pathway in mechanisms of fatty liver, reflected in increased plasma 3-HIB concentrations, and present possible targets for therapeutic intervention.

**Funding:**

Funding was provided by the Research Council of Norway (263124/F20), the University of Bergen, the Western Norway Health Authorities, Novo Nordisk Scandinavia AS, the 10.13039/100016190Trond Mohn Foundation and the 10.13039/501100009707Norwegian Diabetes Association.

**Research in context**

**Evidence before this study**

Fatty liver is strongly correlated with obesity, insulin resistance and type 2 diabetes, but the underlying cellular mechanisms are incompletely understood. A marked characteristic of these interlinked metabolic conditions is elevated circulating concentrations of branched-chain amino acid (BCAA). This elevation of BCAAs may reflect pathogenic cellular changes in BCAA catabolism in key metabolic tissues. However, specific steps in BCAA catabolism that are perturbed in these conditions, and which tissues are involved, remain largely unknown. Recent studies have shown that plasma 3-HIB, an intermediary catabolite of the BCAA valine, which is formed via the enzyme HIBCH, is strongly elevated in insulin resistance, and that 3-HIB modulates lipid uptake and storage in muscle and adipose tissue. Determining a causal role for specific BCAA catabolic pathways in hepatocytes could provide new detailed mechanistic insight into the pathogenesis of fatty liver and lifestyle diseases.

**Added value of this study**

The present study of human hepatocytes uncovers a causal role of the valine/3-HIB pathway in hepatic lipid storage, providing new explanations for the elevated BCAA concentrations in insulin resistance-related conditions. The study comprehensively outlines the implicated transcriptomic and metabolic pathways and networks, and offers data sources for further exploration of genes and metabolites in fatty liver development. The human relevance is supported by targeted gene expression analyses of liver biopsies and plasma metabolite concentrations in patient cohorts.

**Implications of all the available evidence**

Our study altogether provides comprehensive human data on the mechanisms of fatty liver development beyond existing animal models. The data provide important insight into the molecular mechanisms of lipid storage in hepatocytes, and may help to establish treatment targets as well as biomarkers that can be used in the detection, prevention and treatment of fatty liver and associated lifestyle diseases.

## Introduction

Fatty liver diseases are tightly linked to systemic insulin resistance and type 2 diabetes (T2D) and other obesity-related conditions.^1^^,^^2^ These diseases include nonalcoholic fatty liver disease (NAFLD) or metabolically associated fatty acid liver disease (MAFLD), and the more progressed non-alcoholic steatohepatitis (NASH) and cirrhosis. Markers for early detection of these conditions could have great clinical value, and metabolomics has here shown to be a promising approach.^3^, ^4^, ^5^, ^6^, ^7^, ^8^ In part, fatty liver is associated with elevated circulating branched-chain amino acid (BCAA) concentrations,^9^, ^10^, ^11^, ^12^ which are thought to arise from decreased BCAA catabolism in key metabolic tissues.^10^ However, BCAA catabolic pathways are complex, and the roles of specific steps and enzymes in BCAA metabolism in the pathogenesis of fatty liver and insulin resistance remain largely unknown. Notably, decreased BCAA flux into the tricarboxylic acid (TCA) cycle in hepatocytes is associated with NAFLD,^13^ and impaired oxidation of BCAAs may generally cause accumulation of incompletely oxidized TCA cycle intermediates, with consequent reductions in fatty acid (FA) oxidation and increased generation of reactive oxygen species (ROS).^14^^,^^15^

Hydroxybutyrates are a group of structurally related small molecules formed via different pathways of nutrient catabolism, some of which have previously been linked to fatty liver.^3^^,^^4^ We and others have reported that elevated circulating concentrations of the valine-derived catabolite 3-hydroxyisobutyrate (3-HIB), dependent on the enzyme 3-Hydroxyisobutyryl-CoA Hydrolase (HIBCH), are strongly associated with the development of insulin resistance, T2D and gestational diabetes.^16^, ^17^, ^18^, ^19^, ^20^, ^21^ The rate-limiting enzymatic conversion by HIBCH of 3-HIB-CoA into free 3-HIB, which without the CoA moiety can leave the cell, may therefore be a key step in BCAA catabolism that influences fatty liver and the risk of associated diseases. Recent evidence supports a HIBCH- and 3-HIB-dependent regulation of lipid accumulation in muscle and adipose tissue, but a causal role in fatty liver directly in hepatocytes remains unknown. In muscle, the release of 3-HIB from myocytes can promote FA uptake and insulin resistance *in vitro*.^21^ Both white and brown adipocytes are also potential sources of circulating 3-HIB, and alterations in *Hibch* expression and/or 3-HIB concentrations influence adipocyte FA uptake, lipid accumulation, mitochondrial respiration, ROS generation and insulin-stimulated glucose uptake.^20^ Moreover, in rats, pharmacologic activation of hepatic mitochondrial FA β-oxidation lowers plasma 3-HIB concentrations, concomitant with a marked upregulation of *Hibch* mRNA specifically in the liver but not adipose tissue or skeletal muscle, and without affecting other measured BCAA enzymes.^22^ These findings point to hepatic HIBCH as a potential key metabolic regulator at a specific step in BCAA catabolism associated with insulin resistance and fatty liver. Of note, HIBCH may also act downstream in the catabolism of leucine and isoleucine, by removing the CoA group from 3-hydroxypropionyl-CoA to form 3-hydroxypropionate (a precursor of acetyl-CoA).^23^^,^^24^

Fatty liver involves a dysregulation of FA metabolism.^25^^,^^26^ Conversion of pyruvate into acetyl-CoA represents a key regulatory step in hepatic lipid storage, largely determined by the pyruvate dehydrogenase kinase 4 (PDK4) which blocks the activity of the pyruvate dehydrogenase complex (PDC).^27^ Thus, hepatic expression of PDK4 is a sensitive marker of FA β-oxidation,^28^ and NAFLD has been found to involve a loss of regulatory flexibility of PDC.^27^^,^^29^ Changes in these mechanisms may compromise metabolic flexibility, impact FA oxidation, and potentially increase the susceptibility to fatty liver disease and insulin resistance.^30^^,^^31^

We hypothesized that perturbation of *HIBCH* expression in human hepatocytes could modulate mechanisms of hepatic lipid accumulation and metabolic flexibility, and may thereby be a causal player in the development of fatty liver. To test this, we here analyzed *HIBCH* expression in human liver samples and performed experiments in cultured human hepatocytes^32^^,^^33^ to investigate possible disease mechanisms. We uncovered a positive correlation between hepatic *HIBCH* mRNA expression and hepatic fat in humans, an inhibitory effect of HIBCH on oxidative phosphorylation and a stimulatory effect on ROS generation as mechanisms linking HIBCH to fatty liver and metabolic disease. Our transcriptome and metabolomics analyses provide a systematic insight into HIBCH-dependent metabolic functions in human hepatocytes, while also generating new hypotheses of fatty liver development.

## Methods

### Human cohorts

Liver samples were obtained from Caucasian males (n = 37) and females (n = 29) with a wide range of body mass index (BMI: 22.7–45.6 kg/m^2^) who underwent open abdominal surgery for Roux-en-Y bypass, sleeve gastrectomy, elective cholecystectomy or explorative laparotomy (“Liver cohort”). With oral glucose tolerance tests, we identified individuals with T2D (n = 26) or normal glucose tolerance (n = 40). Methods of the phenotypic characterization were described previously.^34^ 3-HIB was analyzed in plasma samples of males (n = 91) and females (n = 101) with obesity (CARBFUNC).^35^ All blood samples were collected between 8 and 11 am after an overnight fast.

### Computed tomography (CT) imaging

Liver density (Hounsfield units, HU) and the volume (cm^3^) of visceral adipose tissue (VAT), subcutaneous adipose tissue (SAT) and total abdominal fat were measured by computed tomography (CT) imaging of the upper abdomen. The upper right diaphragm to vertebral corpus L5/S1 was scanned using a 384-slice multidetector CT scanner (SOMATOM Force, Siemens; Siemens CARE Dose 4D automatic exposure control system; 120 peak kilovoltage; 20 mA). Liver/spleen attenuation index was calculated for internal normalization of liver density, and adipose tissue volumes were determined in the software iNtuition (TeraRecon Inc., San Mateo, CA, USA) (segmentation of pixels with HU values from −195 to −45).^36^ From the participant's upper right diaphragm to the L5/S1 level, the segmentations were done on 5 mm thick cross-sectional CT scan images. Liver and spleen density were calculated as the average HU score per 15 mm^2^ regions of interest (ROI).

### Human hepatocyte cultures

The human liver-derived cell line, Huh7, was grown in Dulbecco's modified Eagle's medium with high glucose (4.5 g/l) containing 10% fetal bovine serum (FBS) (Invitrogen 10106–169) and 1% penicillin and streptomycin (PEST) (Invitrogen 15140–122). The human liver-derived cell line, HepG2, was grown in EMEM Eagle EBSS medium (Lonza ECM0728L) with 10% FBS, 1% PEST and 1% l-Glutamine. Although both Huh7 and HepG2 are well-characterized and commonly used cell lines, they were not further validated against primary human hepatocytes in the present study, nor recently tested for mycoplasma. The cells were grown to 70–80% confluence before 24 h treatments and/or transfections in a serum-free medium. Palmitic acid (PA) (P5585, Sigma–Aldrich) and oleic acid (OA) (O1008, Sigma–Aldrich) were dissolved in 100% EtOH (40 mM and 200 mM stock, respectively). On the day of treatment, these FA-EtOH-stock solutions were dissolved in serum-free DMEM with 1% FA-free BSA and warmed at 37 °C for 1 h to give them time to conjugate. The PDK4 inhibitor 2 ((2,4-dihydroxyphenyl)sulfonyl) isoindoline-4,6-diol (PS10) (SML2418, Sigma-Aldrich)^37^ was dissolved in DMSO (6 mM stock). (±)-Sodium β-hydroxyisobutyrate (3-HIB) (36105, Sigma–Aldrich) was dissolved in water (400 mM stock). Cells were transfected with human *HIBCH* expression plasmid (NM_014362, Human Tagged ORF Clone (RC209814, OriGene)). Knockdown was conducted using small interfering RNA (siRNA) (SASI_Hs01_00064760, Sigma–Aldrich) as described previously.^20^

### Gene expression and functional analyses

Gene expression in liver samples (1 μg RNA input) was analyzed by qPCR using the TaqMan assay as described previously^34^ with target-specific probes (*HIBCH*: Hs00961835_g1; *PDK4*: Hs01037712_m1; *HPRT1*: Hs01003267_m1). For cell cultures, qPCR and RNA sequencing were performed as described previously.^38^ Primer sequences for qPCR with SYBR Green were as follows: F:TGACCTTGATTTATTTTGCATACC, R:CGAGCAAGACGTTCAGTCCT (*HPRT1*); F:TGGTTCTTGCCAGAAACCTTATG, R:GTAGCCACTCGAAATTGCCCA (*HIBCH*); F:AGGACGCGTTTCCAAGTTC, R:TTTATTTGTCTCCCCGCACT (*PDK4*).

For RNA sequencing, 50 ng/μl purified high-quality RNA was used as input and prepared for sequencing and collated into differentially expressed genes as described previously.^38^ Differentially expressed genes were analyzed via PANTHER (http://www.pantherdb.org/(v.14.0)), via the fgsea R package using gene sets derived from the Molecular Signatures Database (MSigDb) and via the R package clusterProfiler.

Oil Red O lipid staining, FA uptake (spectrophotometric assay (MAK156, Sigma–Aldrich)), ROS generation (spectrophotometric assay using a fluorescent probe sensitive to all forms of ROS (CM-H2DCFDA, Thermo Fisher Scientific)) and mitochondrial respiration (Seahorse XF Cell Mito Stress Test Kit, Agilent Technologies) were measured as previously described.^20^ For the FA uptake assay, Huh7 cells (50,000 cells/well) were cultured at 37 °C in a black 96-well flat-bottom plate with clear bottom. On the day of analysis (24 h after transfections/treatments in serum-free medium), the medium was replaced by 100 μl/well of freshly made Fatty Acid Dye Loading Solution (consisting of TF2-C12 Fatty Acid Stock Solution in assay buffer from the kit), according to the manufacturer's protocol. Uptake of the fluorescent dodecanoic (lauric) acid was measured spectrophotometrically on a fluorescence microplate reader at 37 °C (Ex/Em = 485/515 nm) immediately at 10 min intervals for 1 h after its addition to the cells (results after 1 h are shown). Data were normalized to cell number per well using Hoechst staining.

### Western immunoblotting

Cultured hepatocytes were lysed in standard radioimmunoprecipitation assay (RIPA) buffer (Thermo Fisher Scientific) containing protease inhibitor (Roche, Sigma–Aldrich) and PhosphoStop (Sigma–Aldrich). Protein was quantified by the colorimetric DC Protein Assay Kit (Bio-Rad Laboratories) and analyzed by SDS-PAGE and immunoblotting (10 μg protein/well in 4–20% TGX gels (Bio-Rad Laboratories)). Membranes were blocked in blocking solution containing dried skimmed milk or 5% BSA and in TBS/PBS-Tween and incubated with primary antibody against PDK4 (1:500; ab110336 (RRID:AB_10863042); Abcam), HIBCH (1:200; HPA036541 (RRID:AB_2675182); Sigma–Aldrich), vinculin (1:5000; ab18058 (RRID:AB_444215); Abcam), p-Akt (1:2000; 4060S (RRID:AB_2315049); Cell signaling), Akt (1:1000; 9272S (RRID:AB_329827); Cell signaling), p-Pyruvate Dehydrogenase (1:1000; 37115 (RRID:AB_2556543); Cell signaling), Pyruvate Dehydrogenase (1:1000; 3205 (RRID:AB_2162926); Cell signaling), and horseradish peroxidase–conjugated secondary antibody against mouse (1:7500; 554002 (RRID:AB_395198); BD Pharmingen) or rabbit (1:10,000; 3546 (RRID:AB_2058484); Pierce). The antibodies were not further validated for the present study, aside from the confirmation of increased and decreased HIBCH expression upon overexpression and knockdown, respectively. Proteins were detected and quantified using a SuperSignal West Femto Maximum Sensitivity Substrate Kit (Thermo Fisher Scientific), Molecular Imager Gel Doc XR (Bio-Rad Laboratories), and Quantity One 1-D Analysis Software (version 4.6.5).

### Metabolite measurements

Targeted metabolites in human fasting plasma and conditioned cell culture medium were analyzed by GC–MS/MS at Bevital AS, Bergen, Norway.^39^ Metabolite appearance and disappearance per hour was calculated by subtracting the concentration in the conditioned medium from the concentration of the unconditioned medium and dividing the value by 24.

### Metabolic modelling

Constraint-based modelling was based on a genome-scale metabolic reconstruction (GEM) of human liver cells (iHepatocytes2322).^40^ We used flux balance analysis (FBA),^41^ performed in the COBRA Toolbox v3.0^42^ using MATLAB R2018a together with the Mosek linear optimizer. To avoid requiring specific information about the cellular priorities of hepatocytes, we considered an ensemble of simulations where each reaction was treated as the objective function requiring maximization, and report results across this ensemble. We use this approach to allow system-wide highlighting of metabolic pathways that may be influenced by HIBCH perturbation, rather than to quantitatively capture precise behavior of different fluxes—as such, we allow all required metabolites to be present from the original model rather than explicitly modelling growth medium. We modelled HIBCH knockdown as a 50% change in maximum flux through the associated reactions.

### Statistical analyses

Statistics were performed using the software GraphPad Prism 8, IBM SPSS Statistics for Windows, version 27, or R (version 3.6.1). Associations of variables in the human cohorts were assessed by Spearman correlation adjusted for age and sex in IBM SPSS Statistics, with and without correction for multiple testing using the Holm method. The relationship between 3-HIB and *HIBCH* mRNA in FA-treated hepatocyte cultures was analyzed by a linear mixed effects model using the lme4 package in R, accounting for the different experimental treatments with random effects. To evaluate significant difference between two groups, two-tailed unpaired t-tests (if normally distributed) or Mann–Whitney test (if not normally distributed) were performed. Ordinary one-way ANOVA (Sidak's test) (normal distribution) or Kruskal–Wallis (Dunn's test) (not normal distribution) was performed to assess significant differences when comparing more than two groups. A significant difference was defined as p < 0.05 and results are expressed as means ± SEM.

### Ethics statement

All the research was conducted in accordance with both the Declarations of Helsinki and Istanbul. The studies were approved by the Ethics committee of the University of Leipzig (Liver cohort: 363-10-13122010) and the Regional Committee in Western Norway (CARBFUNC: 2017/621/REC West). All participants gave written informed consent before participation.

### Data deposition

The RNA sequencing data are deposited in the European Nucleotide Archive (ENA) with accession number PRJEB59190. The metabolite raw data generated in the experiments in Huh7 cells are included as a supplementary spreadsheet (Supplementary Dataset 1). The flux balance analysis code is available at https://github.com/RoClaGla/metabolic-modeling-HIBCH. However, restrictions apply to the general availability of the human/clinical data because of our ethical approval, patient agreements and the data's sensitive nature. No applicable resources were generated or analyzed during the study.

### Role of the funding source

The funders had no role in study design, data collection, analysis, or interpretation and in the decision to submit the paper.

## Results

### Association of hepatic *HIBCH* expression and plasma 3-HIB with liver fat

**Fig. 1:**
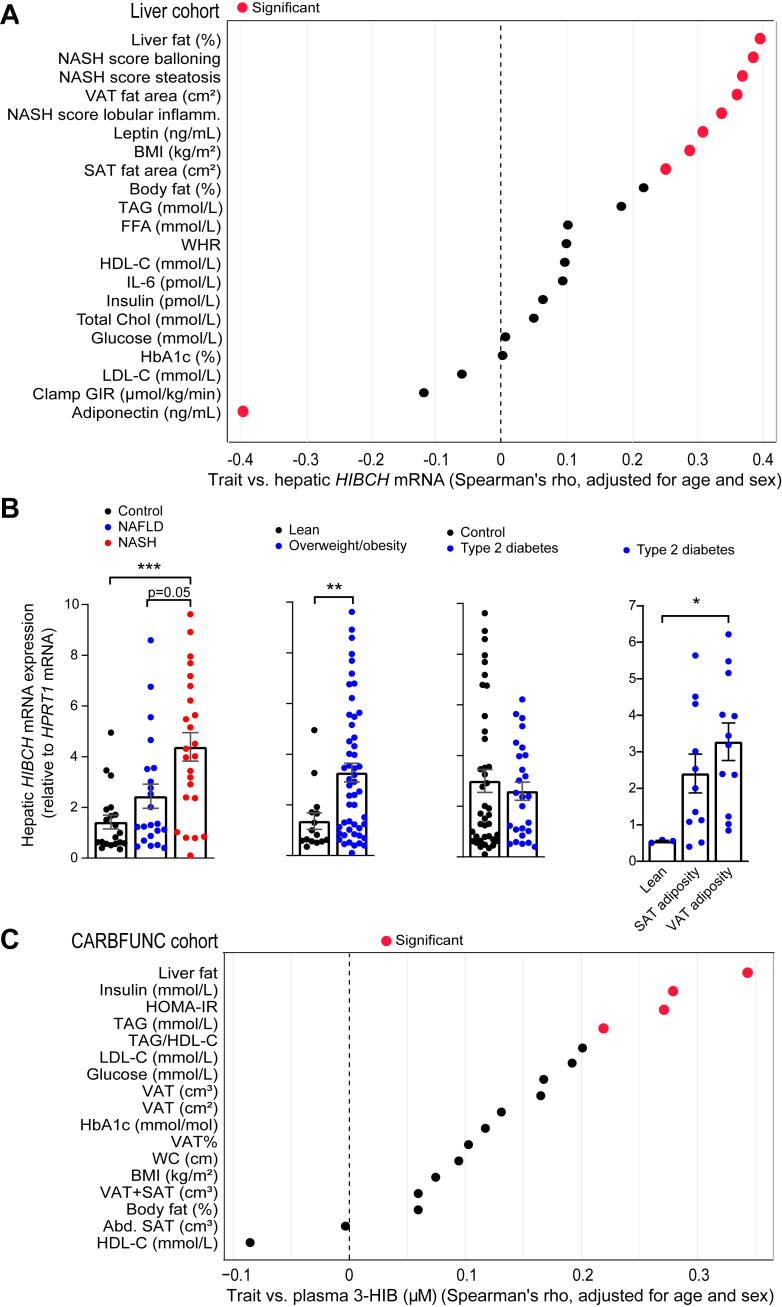
**Hepatic *HIBCH* mRNA expression and plasma 3-HIB concentrations are associated with fatty liver**. A: Graphical representation of Spearman correlations for hepatic *HIBCH* mRNA and different variables in 66 liver donors with different degrees of liver fat content and known NAFLD/NASH status ranging in BMI from 23 to 46 kg/m^2^ (Liver cohort). Correlations are significant for p < 0.05 (indicated by red outline for the analysis without adjustment for multiple testing). B: Hepatic *HIBCH* mRNA expression in people from the Liver cohort. Participants were stratified into different groups based on NAFLD/NASH status, BMI, T2D status and SAT and VAT adiposity. C: Graphical representation of Spearman correlations for plasma 3-HIB and different variables in 192 participants with abdominal obesity (BMI ≥ 30 kg/m^2^) and/or WC ≥ 102 cm (for males) and WC ≥ 88 cm (for females) (CARBFUNC cohort). Liver density was measured by CT imaging and calculated as HU units and divided by spleen density. Because increased liver density reflects lower fat content, the correlation coefficient in the figure was inverted to a positive value (i.e., reflecting more liver fat). Abd. SAT, abdominal subcutaneous adipose tissue; Clamp GIR, glucose infusion rate from euglycemic hyperinsulinemic clamp; FFA, free fatty acids; HDL-C, high-density lipoprotein cholesterol; IL-6, interleukin-6; LDL-C, low-density lipoprotein cholesterol; SAT, subcutaneous adipose tissue; VAT, visceral adipose tissue; TAG, triacylglycerols; WC, waist circumference; WHR; Waist-Hip-Ratio.∗p < 0.05, ∗∗p < 0.01, ∗∗∗p < 0.001 (One-way ANOVA–Sidak's test, Kruskal–Wallis—Dunn's test or Mann–Whitney test).

In a cohort of individuals with different degrees of adiposity (Liver cohort), liver *HIBCH* mRNA expression was most strongly correlated to liver fat percentage, non-alcoholic steatohepatitis (NASH) score and VAT area (positive correlations), and serum adiponectin concentrations (negative correlation) (Fig. 1A). Among additional clinical features that less precisely reflect liver fat, there were less marked positive correlations between liver *HIBCH* mRNA and serum leptin, SAT area, BMI and total body fat percentage (Fig. 1A). After correction for multiple testing of all the analyzed variables, significant correlations were only seen for liver fat percentage (p = 0.024), NASH (p = 0.032) and adiponectin (p = 0.034). Moreover, liver *HIBCH* mRNA expression was significantly elevated in those with NASH and obesity compared to those without (Fig. 1B). These data show a particular correlation between hepatic *HIBCH* expression and liver fat, and weaker correlations for other clinical features in metabolic syndrome.

In our second cohort (CARBFUNC) where metabolite but not liver biopsy data were available, we found a positive correlation between circulating 3-HIB concentrations and liver fat (p < 0.001) as well as with insulin, HOMA-IR and triacylglycerols (TAG) (p < 0.05) (Fig. 1C), with significant correlations also after correction for multiple testing. Similar significant correlations were observed for valine (Supplementary Fig. S1A). For an expanded set of metabolites measured in CARBFUNC, 3-HIB was among the most strongly correlated metabolites with liver fat but not with BMI (Supplementary Fig. S1B and C, Supplementary Table S2). Other metabolites significantly correlated with liver fat included α-ketoglutarate, α-hydroxybutyrate, cysteine, all BCAAs and acetoacetate (positive correlation) and glycine and serine (inverse correlation) (Supplementary Fig. S1B, Supplementary Table S2). α-ketoglutarate, cysteine and acetoacetate also showed a positive and glycine an inverse correlation with BMI (Supplementary Fig. S1B, Supplementary Table S2).

### Lipid-storing hepatocytes increase *HIBCH* expression and release 3-HIB

**Fig. 2:**
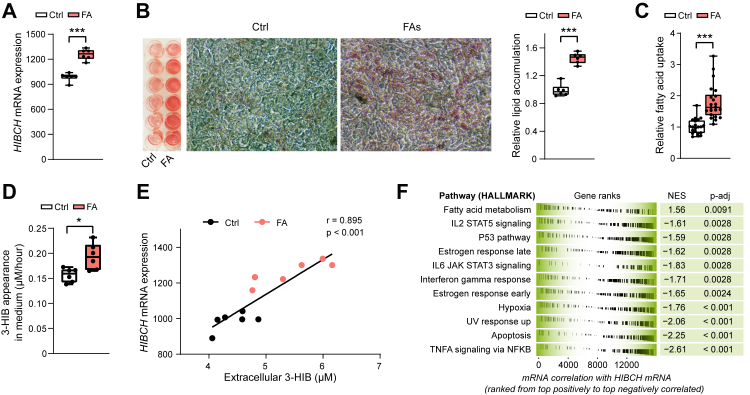
**Lipid storage in hepatocytes is accompanied by increased *HIBCH* expression and release of 3-HIB**. Huh7 liver cells were treated with and without free FAs (1:1 M ratio of 50 μM PA and 50 μM OA) for 24 h before analyses. Gene expression was measured by RNA-sequencing. A: *HIBCH* expression in control and FA-treated cells, shown as RPKM (n = 6). B: Representative images of Oil-Red-O lipid-stained hepatocytes (left) and quantification of lipid accumulation (right) in Huh7 cells (n = 6). C: Relative FA uptake in Huh7 cells treated with or without FA for 24 h, followed by addition of fluorescent dodecanoic acid whose uptake was detected spectrophotometrically after 1 h (n = 9–12). D: Average net medium appearance per hour of 3-HIB during a 24 h period (n = 6). E: A linear mixed model accounting for the group structure was used to examine the correlation between *HIBCH* mRNA expression (RPKM) and extracellular 3-HIB concentrations (GC–MS/MS analysis of cell culture medium) across the control and FA-treated samples. F: GSEA showing up- and down-regulated pathways by *HIBCH* expression (HIBCH correlation) in Huh7 (HALLMARK pathway analysis) (n = 6). Gene sets are ordered by normalized enrichment score (NES) and significant adjusted p-value for each pathway are shown. Ctrl, control; FA, fatty acids; GSEA, gene set enrichment analysis; RPKM, reads per kilobase per million mapped reads.∗p < 0.05, ∗∗p < 0.01, ∗∗∗p < 0.001 (Unpaired t-test or Mann–Whitney test).

Human Huh7 hepatocyte cultures were treated with PA and OA (1:1 ratio) for 24 h to induce hepatocyte lipid accumulation, as a model of fatty liver,^43^ using a low FA dose (100 μM) to prevent lipotoxicity.^44^ There were no significant differences in lipid accumulation between different FA doses (Supplementary Fig. S2A). *HIBCH* mRNA expression was increased in the lipid-storing hepatocytes compared to controls (Fig. 2A and B). Increased FA uptake was also confirmed by measuring the uptake of a fluorescent FA substrate (Fig. 2C). 3-HIB appearance in the medium increased from the lipid-storing hepatocytes (Fig. 2D), and *HIBCH* mRNA correlated positively to the extracellular 3-HIB concentrations (Fig. 2E).

Combining RNA-sequencing data for samples treated with and without FA, Gene Set Enrichment Analysis (GSEA) showed that *HIBCH* mRNA correlated positively with mRNA expression for several genes involved in FA metabolism and negatively to pathways including TNF signalling via NF-κB (Fig. 2F). Moreover, PANTHER gene ontology analysis revealed positive correlations between *HIBCH* mRNA and mRNA for genes involved in, e.g., FA oxidation, NADPH binding (linked to processes in the TCA cycle) and histidine catabolic process (10-, 33- and 45-fold enrichment, respectively), and a negative correlation with mRNA for genes that respond to hydrogen peroxide (a form of ROS) (6-fold enrichment) (Supplementary Fig. S2B).

A volcano plot of the global transcriptome changes upon FA treatment revealed *PDK4* as the most upregulated gene (Supplementary Fig. S2C). Additionally, genes encoding metallothioneins (*MT1E, MT1F, MT2A*) and *AKR1C2* were among those with the strongest downregulation (Supplementary Fig. S2C). As a further illustration of the degree of change among the top regulated genes, FA treatment in Huh7 cells increased and decreased the expression of *PDK4* and *AKR1C2* by 3- and 6-fold, respectively (Supplementary Fig. S2D), and the expression of these genes was inversely correlated with that of *HIBCH* (Supplementary Fig. S2E). Experiments in another human hepatocyte cell line, HepG2, confirmed FA-induced lipid accumulation (Supplementary Fig. S2F) as well as upregulation of both *HIBCH* and *PDK4* mRNA (Supplementary Fig. S2G).

When analyzing the effect of FA treatment *per se* by GSEA, we found marked gene enrichment in, e.g., oxidative phosphorylation, bile acid metabolism, FA metabolism and glycolysis (upregulated) and TNF signalling via NF-κB and hypoxia (downregulated) (Supplementary Fig. S3A). An upregulation of oxidative phosphorylation by FA treatment was supported by increased oxygen consumption rates and calculated basal respiration, ATP synthesis, maximal respiration and proton uncoupling, measured in living Huh7 cells (Supplementary Fig. S3B), but without change in the generation of ROS (Supplementary Fig. S3C).

### HIBCH overexpression alters lipid- and energy metabolism in hepatocytes

**Fig. 3:**
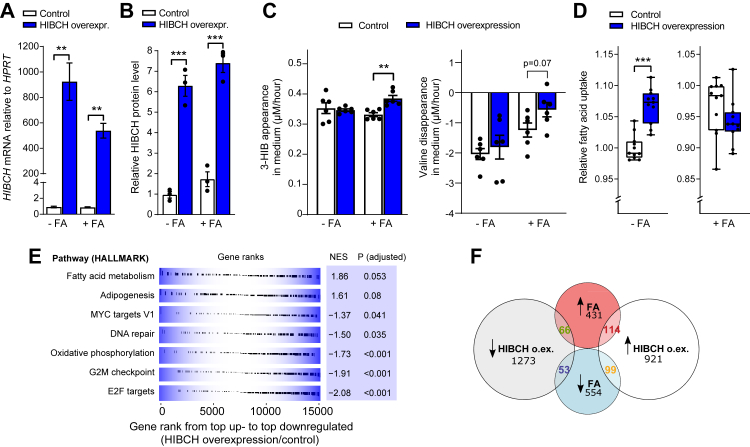
***HIBCH* overexpression in hepatocytes affects cellular pathways related to FA metabolism and oxidative phosphorylation**. Huh7 liver cells were transfected with pCMV6-HIBCH or control (pCMV6-empty vector) plasmid (0.2 μg per well in a 24-well plate) diluted in Opti-MEM® Reduced Serum Media and TransIT-X2® transfection reagent (Mirus). 24 h after transfection, the cells were treated with and without free FAs (1:1 M ratio of 50 μM PA and 50 μM OA) for 24 h before analyses. A: *HIBCH* expression in Huh7 cells measured by qPCR (n = 6). B: The quantitative values of HIBCH relative to α-VINCULIN in Huh7 cells (n = 3). C: Average net medium appearance per hour of 3-HIB and valine during a 24 h period (n = 6). D: Relative FA uptake in Huh7 cells treated with or without FA for 24 h, followed by addition of fluorescent dodecanoic acid whose uptake was detected spectrophotometrically after 1 h (n = 9–12). E: GSEA showing up-and down-regulated pathways by HIBCH overexpression relative to control expression (without and with FAs) in Huh7 (HALLMARK pathway analysis of RNA sequencing data) (n = 24). Gene sets are ordered by normalized enrichment score (NES) and significant adjusted p-values for each pathway are shown. F: Number of up-and down-regulated genes by HIBCH overexpression with/without FA treatment in Huh7 cells shown as a Venn diagram (adjusted p-value cutoff <0.1). FA, fatty acid treatment; NES, normalized enrichment score.∗p < 0.05, ∗∗p < 0.01, ∗∗∗p < 0.001 (–unpaired t-test or Mann–Whitney test).

HIBCH overexpression (increasing *HIBCH* mRNA by 600-900-fold (Fig. 3A) and HIBCH protein by ∼5-fold (Fig. 3B, Supplementary Fig. S4A)) increased the net 3-HIB appearance in the culture medium when combined with FA treatment, and this appeared to correspond to a lower cellular uptake of valine (Fig. 3C). Although this response appears only moderate on the short timescale of the experiment, its effect over time may well be more substantial. The overexpression also stimulated FA uptake in cells not treated with FAs (Fig. 3D). A volcano plot (excluding *HIBCH* as well as *GH1* due to their high expression changes/low p-values) revealed several genes that were strongly affected by *HIBCH* overexpression (Supplementary Fig. S4B), including *IPCEF1* and *NR1D1* among the upregulated genes and *FGFR1* and *GH1* among the downregulated genes (Supplementary Fig. S4C). When analyzing the effect of HIBCH overexpression by GSEA, several upregulated genes were found to be involved in FA metabolism/lipid storage, while downregulated genes were strongly enriched in categories related to cell cycle/proliferation and replication and repair of DNA and mitochondria (i.e., E2F targets, G2M checkpoint, DNA repair) as well as oxidative phosphorylation (Fig. 3E). Among the 2194 genes significantly affected by HIBCH overexpression, 332 were also up- or downregulated by FA treatment (Fig. 3F). HIBCH overexpression and FA treatment had either the same or opposite effects on the expression of these genes in four distinct patterns (Supplementary Fig. S4D). These data demonstrate that increased *HIBCH* expression has wide-reaching effects on the hepatocyte transcriptome, including downregulation of genes that promote cellular respiration, replication and repair.

### Transcriptome effects of HIBCH knockdown

**Fig. 4:**
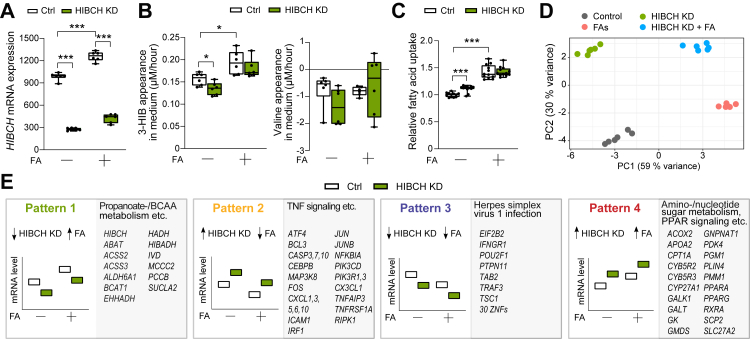
**HIBCH knockdown and FA treatment in hepatocytes have both similar and distinct metabolic effects**. Huh7 liver cells were treated with and without free Fas (1:1 M ratio of 50 μM PA and 50 μM OA) combined with siRNA-mediated knockdown of HIBCH and siRNA non-targeting control for 24 h before analyses. A: *HIBCH* expression in Huh7 cells measured by mRNA sequencing (n = 6). B: Average net medium appearance per hour of 3-HIB and valine during a 24 h period (n = 6). C: Relative FA uptake in Huh7 cells treated with or without FA for 24 h, followed by addition of fluorescent dodecanoic acid whose uptake was detected spectrophotometrically after 1 h (n = 9–12). D: Principal component (PC) analysis of samples in all four treatment groups in the experiment are shown. E: Representative functional categories and genes in the four different gene expression patterns responding to FA treatment and HIBCH knockdown. FA, fatty acid treatment; KD, knockdown.∗p < 0.05, ∗∗P < 0.01, ∗∗∗p < 0.001 (Ordinary one-way ANOVA–Sidak's test).

Opposite to overexpression, HIBCH knockdown (80% reduction of *HIBCH* mRNA levels (Fig. 4A) and 25–50% reduction of HIBCH protein (Supplementary Fig. S5A)) reduced 3-HIB release from the hepatocytes in cells not treated with FA (Fig. 4B). This decrease in 3-HIB was however not observed in cells treated with FA (Fig. 4B) even though knockdown completely counteracted an FA-induced increase in HIBCH protein (Supplementary Fig. S5A). We observed no change in net consumption of valine by the treatments (Fig. 4B). Surprisingly, similar to HIBCH overexpression, HIBCH knockdown increased the relative FA uptake in cells not exposed to excess FAs (Fig. 4C).

Principal component analysis (PCA) of the RNA sequencing data showed that principal component 1 (PC1) explained 59% and principal component 2 (PC2) 30% of the variance when comparing the effects of both FA and HIBCH knockdown (Fig. 4D). Thus, most of the variance (89%) was explained by these components. The transcriptome effect of FA treatment dominated PC1, but HIBCH knockdown modulated this effect along PC1 as well as clearly separating the samples in PC2 (Fig. 4D). A volcano plot revealed numerous genes that were strongly and consistently affected by HIBCH knockdown regardless of FA treatment, including *KLHL2*, *NIPA1*, *NCOA7*, *CCN2*, *SERPINE1*, *AKR1C1* and *AKR1C2* among the upregulated genes and *NUDT15*, *NEK7*, *METAP1*, *NOTCH2* and *GSN* among the downregulated genes (Supplementary Fig. S5B).

In total, HIBCH knockdown affected 2727 genes that were also up- or downregulated by FA treatment (Supplementary Fig. S5C), showing either the same or opposite expression in four distinct patterns (Fig. 4E, Supplementary Fig. S5D). GSEA for all samples (treated with or without FA) showed a predominant effect of HIBCH knockdown on genes related to TNF signalling via NF-κB and hypoxia (upregulation) (Supplementary Fig. S6A), consistent with an inverse relationship between *HIBCH* expression and TNF signalling. For the FA-dependent patterns, Pattern 1 (downregulated by knockdown and upregulated by FA treatment, following the *HIBCH* expression) included genes involved in propanoate (i.e., propionate) metabolism and BCAA degradation (Fig. 4E, Supplementary Fig. S6B). Within these pathways, in which HIBCH participates, HIBCH knockdown downregulated several genes downstream of HIBCH, including *HIBADH*, *EHHADH* and *PCCB* (Supplementary Fig. S6C). In contrast, several genes upstream of HIBCH in these pathways showed an upregulation upon *HIBCH* knockdown, including *BCKDHA*, *BCKDHB*, *DLD* and *DBT*, possibly reflecting a metabolic and/or transcriptional feedback loop or compensatory mechanisms. Pattern 2 (upregulated by knockdown and downregulated by FA treatment, opposite to *HIBCH* expression) included genes involved in circadian rhythm and TNF signalling (Supplementary Fig. S6B). In addition to *FOS*, *JUN* and *TNFRSF1A*, chemokines (e.g., *CXCL* family) and other immediate-early genes (Fig. 4E, Supplementary Fig. S6C), the HIBCH-dependent genes related to TNF signalling included important metabolic regulators such as *CEBPB*, *ZFP36*, *SERPINE1*, *CCN2*, *HMGCS1* and *NAMPT* (Supplementary Fig. S6C). Pattern 3 (downregulated by knockdown and FA treatment) was characterized by genes related to herpes simplex virus infection (Fig. 4E), as well as several genes involved in energy and lipid metabolism such as *AKR1D1*, *HCCS* and *ADIPOR2* (Supplementary Fig. S6C). Finally, pattern 4 (upregulated by knockdown and FA treatment) included regulators of FA and mitochondrial metabolism such as *CPT1A*, *CYCS* and *PDK4* (Supplementary Fig. S6C), and other genes involved in amino sugar and nucleotide metabolism, inositol phosphate metabolism and PPAR signalling (Fig. 4E, Supplementary Fig. S6B).

### Effect of HIBCH knockdown on insulin signalling

Previous studies of myocytes^21^^,^^45^ and adipocytes^20^ reported an inhibitory effect of 3-HIB on insulin-stimulated glucose uptake, involving reduced AKT phosphorylation.^45^ We therefore applied GSEA to see if increased or decreased HIBCH expression affected genes involved in insulin signalling in the Huh7 hepatocytes. When combining the samples with and without fatty acid treatment, the gene set “insulin receptor signalling cascade” was significantly affected both by HIBCH overexpression and knockdown (adjusted enrichment p = 0.034 and p = 0.021, respectively). Several genes in this pathway were regulated in opposite directions by HIBCH overexpression and knockdown, including *IRS2*, *MAPK2*, *HRAS*, *FGFR1*, *FGFR2* and *FGF18* which were upregulated by knockdown and downregulated by overexpression, and *GRB10* and *PIK3CA* which were downregulated by knockdown and slightly upregulated by overexpression (Supplementary Fig. S7A). To further assess if insulin signalling was affected, we measured phosphorylated Akt by Western blotting and found increased Akt phosphorylation upon HIBCH knockdown in the Huh7 cells without FA treatment (Supplementary Fig. S7B), supporting that HIBCH can attenuate insulin signalling in hepatocytes. FA treatment strongly suppressed Akt phosphorylation, and in this condition HIBCH knockdown was unable to restore the phosphorylation (Supplementary Fig. S7B).

### Regulatory relationship between HIBCH and PDK4

**Fig. 5:**
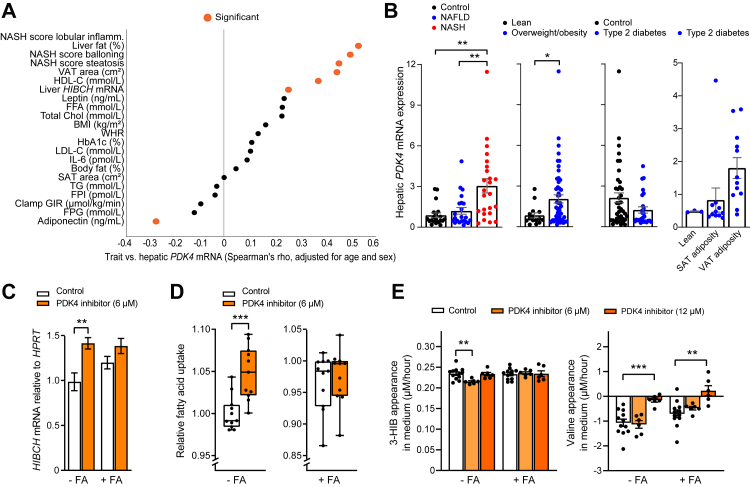
**HIBCH and 3-HIB are responsive to PDK4 inhibition**. A: Graphical representation of Spearman correlations for hepatic *PDK4* mRNA and different variables in 66 liver donors with different degrees of liver fat content and known NAFLD/NASH status ranging in BMI from 23 to 46 kg/m^2^ (Liver cohort). Correlations are significant for p < 0.05 (indicated by black outline for the analysis without adjustment for multiple testing). B: Hepatic *PDK4* mRNA expression in participants from the Liver cohort. Participants were stratified based on NAFLD/NASH status, BMI, T2D status and SAT and VAT adiposity. C–E: Huh7 liver cells were treated with and without free FAs (1:1 M ratio of 50 μM PA and 50 μM OA) combined with PDK4 inhibitor (final concentration of 6 or 12 μM PS10) (DMSO was used as control) for 24 h before analyses. C: *HIBCH* mRNA expression in Huh7 cells measured by qPCR, calculated relative to the reference gene *HPRT* (n = 6). D: Effect of PDK4 inhibitor on relative FA uptake in Huh7 cells treated with or without FA for 24 h, followed by addition of fluorescent dodecanoic acid whose uptake was detected spectrophotometrically after 1 h (n = 9–12). E: Average net medium appearance per hour of 3-HIB and valine in Huh7 cells during a 24 h period (n = 6) in response to PDK4 inhibitor. Clamp GIR, glucose infusion rate from euglycemic hyperinsulinemic clamp; FA, fatty acid treatment; FFA, free fatty acids; HDL-C, high-density lipoprotein cholesterol; IL-6, interleukin-6; LDL-C, low-density lipoprotein cholesterol; SAT, subcutaneous adipose tissue; VAT, visceral adipose tissue; TAG, triacylglycerols.∗p < 0.05, ∗∗p < 0.01, ∗∗∗p < 0.001 (Ordinary one-way ANOVA–Sidak's test, unpaired t-test, Kruskal–Wallis—Dunn's test or Mann–Whitney test).

Underscoring the relevance of PDK4 in fatty liver disease,^29^^,^^46^ and in line with the upregulation of both *PDK4* and *HIBCH* mRNA expression in cultured hepatocytes treated with FAs (Fig. 2A, Supplementary Fig. S2G), in patient liver samples we found strong positive correlations between hepatic *PDK4* mRNA expression and several features of fatty liver disease (Fig. 5A), and these correlations remained significant after correction for multiple testing. Like hepatic *HIBCH* mRNA (Fig. 1A), hepatic *PDK4* mRNA showed a significant negative correlation with adiponectin (Fig. 5A) (although not significant after multiple testing). Hepatic *PDK4* and *HIBCH* mRNA levels were also significantly positively correlated (Fig. 5A), and, like *HIBCH* mRNA (Fig. 5B), hepatic *PDK4* mRNA was significantly elevated in people with NASH (Fig. 5B).

Increased *PDK4* expression in livers of people with fatty liver disease could be a compensatory mechanism to upregulate FA oxidation.^25^ This compensatory role of PDK4 may contrast with a possible primary role of HIBCH in fatty liver, since HIBCH knockdown increased *PDK4* mRNA expression in the Huh7 hepatocytes (Fig. 4E, Supplementary Fig. S6C). The PDK4 upregulation upon HIBCH knockdown was supported by observations in HepG2 hepatocytes (Supplementary Fig. S8A) and confirmed on the protein level in the Huh7 cells not treated with FA (Supplementary Fig. S8B). FA treatment strongly increased PDK4 protein, and HIBCH knockdown did not increase the level further (Supplementary Fig. S8B), possibly due to a saturation of PDK4 protein. We additionally assessed if the increased PDK4 expression was functionally reflected in increased inhibitory phosphorylation of its target pyruvate dehydrogenase complex (PDC), which when activated converts pyruvate to acetyl-CoA. Supporting increased PDK4 activation by HIBCH knockdown, we observed an increased PDC phosphorylation by up to 50% (with an attenuated effect in the hepatocyte cultures treated with FA) (Supplementary Fig. S8B).

To further establish the existence of a negative feedback loop between *PDK4* and *HIBCH*, we examined if PDK4 inhibition would increase *HIBCH* mRNA expression and thereby the potential for lipid storage. Strikingly, addition of the selective PDK4 inhibitor PS10^37^ to the Huh7 hepatocytes increased *HIBCH* mRNA by 50% (Fig. 5C), and this was accompanied by increased FA uptake in the cells that were not treated with FAs (Fig. 5D). In contrast, PDK4 inhibition decreased net release of 3-HIB despite the increased *HIBCH* mRNA expression and despite no change in valine consumption (while the higher dose of PS10, which did not affect net 3-HIB release or FA uptake, nullified the otherwise notable valine consumption) (Fig. 5E).

### Altered hepatocyte *HIBCH* expression affects mitochondrial respiration and extracellular metabolite concentrations

**Fig. 6:**
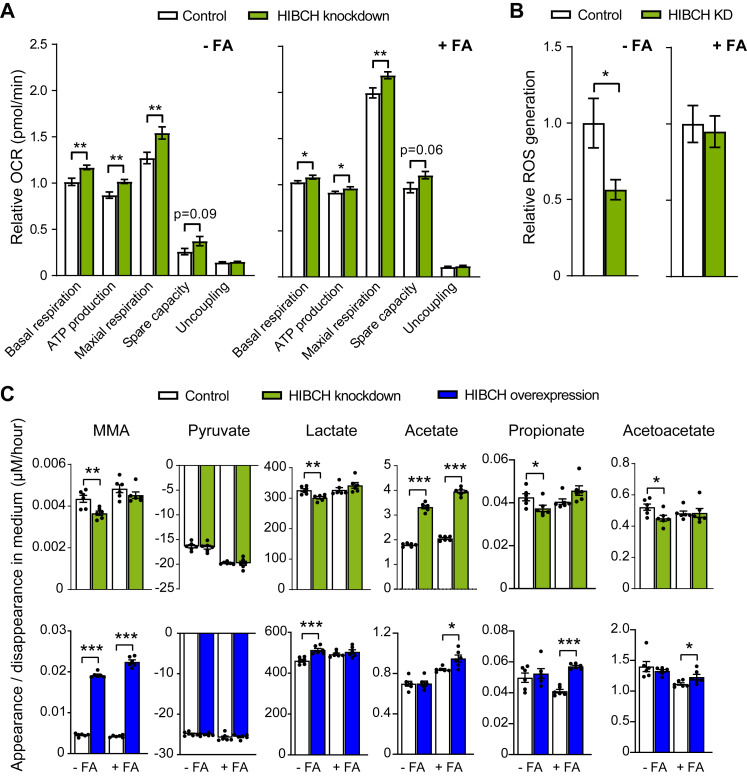
**Altered *HIBCH* expression affects mitochondrial respiration, ROS production and extracellular metabolite concentrations**. Huh7 liver cells were transfected with siRNA-mediated knockdown of HIBCH and siRNA non-targeting control or with pCMV6-HIBCH or control (pCMV6-empty vector) plasmid (0.2 μg per well in a 24-well plate) diluted in Opti-MEM® Reduced Serum Media and TransIT-X2® transfection reagent (Mirus). The cells were treated with and without free FAs (1:1 M ratio of 50 μM PA and 50 μM OA) for 24 h, before analyses. A: Seahorse Cell Mito Stress Assay (OCR measurements) was performed using the Seahorse XFe96 Analyzer to assess the mitochondrial respiration in Huh7 cells (n = 10–12) Basal levels (the three first OCR measurements) were obtained, before adding oligomycin, CCCP and rotenone/antimycin A. Basal respiration, ATP production, maximal respiration, spare capacity and uncoupling were calculated for each well based on the OCR measurements. B: Effect of HIBCH knockdown on ROS production in Huh7 cells treated with or without FA for 24 h, followed by addition of fluorescent probe whose uptake was detected spectrophotometrically after 1 h (n = 11). C: Average net medium appearance per hour of the metabolites during a 24 h period (n = 6). FA, fatty acid; KD, knockdown; OCR, oxygen consumption rate.∗p < 0.05, ∗∗p < 0.01, ∗∗∗p < 0.001 (unpaired t-test).

To directly test if reduced *HIBCH* expression increases mitochondrial respiration, consistent with the increased expression of *PDK4* and *CPT1A* upon HIBCH knockdown (Supplementary Fig. S6C), we measured HIBCH-dependent oxygen consumption rate (OCR) in live Huh7 hepatocytes. We found that knockdown significantly increased mitochondrial respiration both in the absence and presence of exogenous FAs (Fig. 6A, Supplementary Fig. S8C), even in the context of an already increased oxygen consumption induced by FA treatment alone (Supplementary Fig. S3B). The calculated basal respiration, ATP synthesis and maximal respiratory capacity were all increased significantly, with a similar tendency for spare capacity but no effect on uncoupling of the respiratory chain (Fig. 6A). Concomitantly, the knockdown decreased ROS levels particularly in cells without FA treatment (Fig. 6B).

**Fig. 7:**
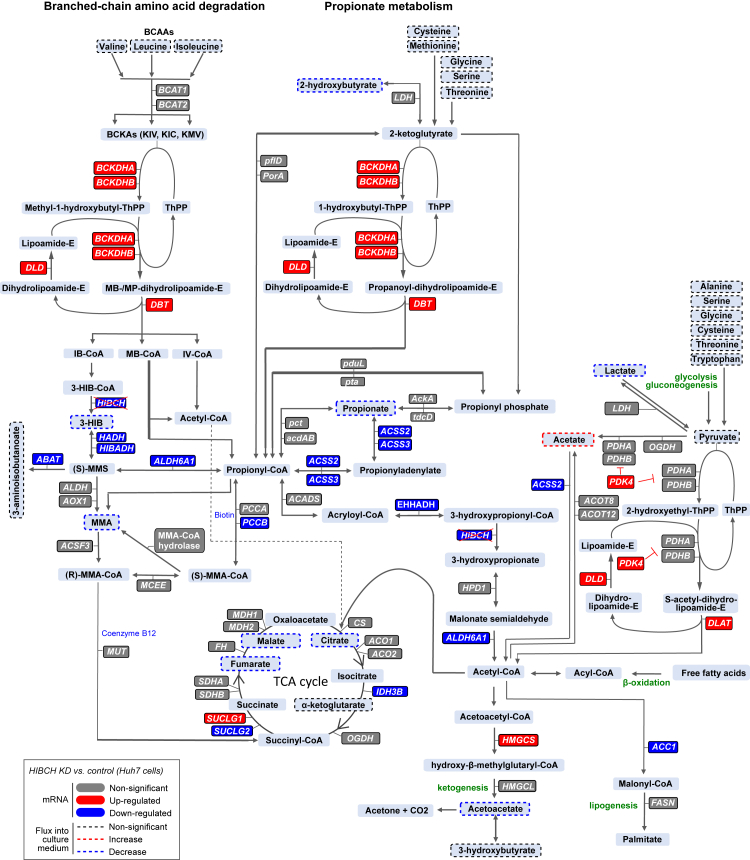
**Overview of BCAA and propionate metabolism and effects of HIBCH knockdown in cultured Huh7 hepatocytes**. Down- and upregulated genes (from RNA-seq) are marked in blue and red colour, respectively. Increased and decreased net medium appearance of metabolites based on extracellular measurements are marked with dotted lines in blue and red colour, respectively. Genes and metabolites that are unchanged by HIBCH knockdown are marked in grey colour and black dotted lines, respectively.

We furthermore measured whether these metabolic changes were reflected in changes in extracellular concentrations of additional intermediary metabolites downstream in BCAA metabolism, as well as in key metabolic substrates including pyruvate, lactate, acetate and ketones (Fig. 6C). MMA, which is partly formed from 3-HIB via methylmalonyl semialdehyde (MMS) (Fig. 7) and which we previously found associated with hepatic *Hibch* expression in rats,^22^ showed a lower appearance in the culture medium upon HIBCH knockdown and a markedly increased appearance upon HIBCH overexpression regardless of free FA addition (Fig. 6C). While pyruvate was not affected, changes were also seen in extracellular concentrations of lactate, showing reduced medium appearance with knockdown and increased appearance with overexpression but only in the absence of additional FA (Fig. 6C). The short-chain fatty acid (SCFA) acetate is another key carbon-harboring metabolite that is not only produced by the gut microbiota but also in the liver and utilized by other peripheral tissues.^47^ Acetate can be derived from glucose via pyruvate^48^ and may interconvert into and from acetyl-CoA (Fig. 7). Interestingly, HIBCH knockdown resulted in a marked increase in extracellular acetate concentrations, both in conditions with and without FA, although also overexpression increased acetate appearance in the medium to some degree in FA-treated cells (Fig. 6C). Propionate, another SCFA whose metabolism is closely linked to BCAA metabolism via their convergence on propionyl-CoA (Fig. 7), showed decreased and increased medium appearance with knockdown and overexpression, respectively (Fig. 6C), consistent with the downregulation of several genes in this pathway that we observed upon HIBCH knockdown (pattern 1, Fig. 4E). As these pathways promote anaplerosis by different routes including via acetyl-CoA, we additionally measured selected TCA cycle metabolites and expression of genes involved in the TCA cycle. We found that HIBCH knockdown lowered the net appearance in the culture medium of fumarate, malate and citrate, specifically in cells that were not treated with FAs, while no changes were seen for α-ketoglutarate (Supplementary Fig. S8D). Conversely, HIBCH overexpression increased the medium appearance of fumarate and malate for cells treated with FA, while no changes were observed for citrate and α-ketoglutarate (Supplementary Fig. S8D). Finally, a similar opposite effect of knockdown and overexpression as on fumarate and malate appearance in the medium was observed for the ketone acetoacetate (Fig. 6C). Considering the pathways as a whole, gene expression patterns upon knockdown suggested an overall downregulation of enzymatic reactions downstream of the 3-HIB formation step, and a compensatory upregulation of genes encoding enzymes upstream of this step (Fig. 7).

### Metabolic modelling points to changes in BCAA metabolism and acyl-carnitine shuttling

To further predict specific metabolic changes upon loss of HIBCH we performed constraint-based metabolic modelling of human liver cells using a set of flux balance analysis simulations with the iHepatocytes model,^40^ treating each internal reaction as a possible objective. The modelling predicted that 34 reactions among the following subsystems were compromised upon HIBCH knockdown: FA β-oxidation, BCAA metabolism, β-alanine metabolism, propanoate metabolism, and mitochondrial carnitine shuttle, transport of MMA and valine to the mitochondria and of L-3-aminobutanoate from the mitochondria, and excretion of valine and 2,6-dimethylheptanoyl-carnitine from the liver cells (Supplementary Table S3). Each of these reactions, when regarded as the objective requiring maximization of metabolic flux, reduced their maximal activity by ∼50% after HIBCH knockdown. Enzymes linked to these reactions include ABAT, ACAA2, ACADL, ACADM, ALDH7XAY, AOX1, BCAT1, BCAT2, BCKDHA, BCKDHB, DBT, ECHS1, EHHADH, HADH, HADHA, HADHB, HSD17B10 and TMEM91 (Supplementary Table S3).

### Addition of 3-HIB affects hepatocyte energy- and lipid metabolism

**Fig. 8:**
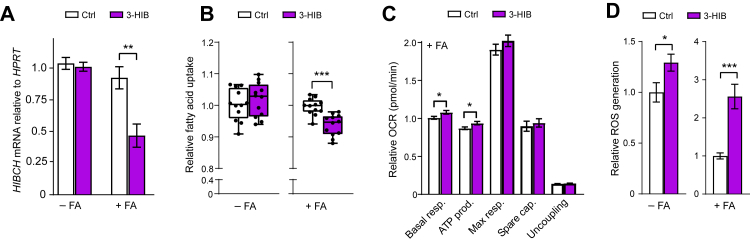
**3-HIB supplementation to human hepatocytes alters key metabolic functions**. Huh7 liver cells were treated with and without free FAs (1:1 M ratio of 50 μM PA and 50 μM OA) combined with and without 3-HIB supplementation (final concentration of 25 μM) (water was used as control), for 24 h before analyses. A: *HIBCH* mRNA expression in Huh7 cells measured by qPCR, calculated relative to the reference gene *HPRT* (n = 6). B: Relative FA uptake in Huh7 cells treated with or without FA for 24 h, followed by addition of fluorescent dodecanoic acid whose uptake was detected spectrophotometrically after 1 h (n = 9–12). C: Seahorse Cell Mito Stress Assay (OCR measurements) was performed using the Seahorse XFe96 Analyzer to assess the mitochondrial respiration in Huh7 (n = 10–12) 24 h after treatment. Basal levels (the three first OCR measurements) were obtained, before adding oligomycin, CCCP and rotenone/antimycin A, as indicated at the top in the upper left figure. Basal respiration, ATP production, maximal respiration, spare capacity and uncoupling were calculated for each well based on the OCR measurements. D: Effect of 3-HIB supplementation on ROS generation in Huh7 cells treated with or without FA for 24 h, followed by addition of fluorescent probe whose uptake was detected spectrophotometrically after 1 h (n = 11). FA, fatty acid treatment.∗p < 0.05, ∗∗p < 0.01, ∗∗∗p < 0.001 (unpaired t-test).

Finally, we investigated if supplementing hepatocytes with physiological doses of 3-HIB affects metabolic processes relevant for fatty liver. First, we assessed if addition of 3-HIB would affect *HIBCH* mRNA expression in a regulatory loop, and found that 3-HIB halved the *HIBCH* mRNA level specifically in hepatocytes treated with FAs (Fig. 8A). 3-HIB also lowered FA uptake in FA-treated cells (Fig. 8B), opposite to the effects observed previously in endothelial cells^17^^,^^21^ and adipocytes,^20^ although it should be noted that the previous studies did not apply FA treatment. Furthermore, as observed for the HIBCH knockdown (Fig. 5A), 3-HIB addition increased basal respiration and ATP production albeit without increasing maximal respiration significantly and only observed in FA-treated cells (Fig. 8C, Supplementary Fig. S9). Lastly, 3-HIB addition increased ROS generation regardless of FA treatment but with the greatest (1.5-fold) increase in FA-treated cells (Fig. 8D).

## Discussion

Altered blood concentrations and cellular catabolism of BCAAs are strongly associated with increased fat storage and insulin resistance, but the tissues and specific causal pathways responsible for these associations remain to be elucidated. In the present study of hepatocytes, we have uncovered important metabolic roles for HIBCH, a hydrolase acting in the valine degradation pathway to form 3-hydroxyisobutyrate as well as in the downstream pathway that converts propionyl-CoA to acetyl-CoA. We found elevated *HIBCH* mRNA in the liver of patients with fatty liver disease and visceral adiposity, and increased *HIBCH* mRNA and extracellular 3-HIB concentrations concomitant with substantial lipid accumulation in cultured human hepatocytes exposed to FAs. HIBCH knockdown and overexpression decreased and increased the medium appearance of 3-HIB, respectively. A key finding is that HIBCH knockdown increased the cellular oxygen consumption rate. Hepatocyte ROS levels were also markedly affected, showing a decrease upon HIBCH knockdown and an increase by addition of 3-HIB. Finally, our data reveal distinct impacts of alterations in the valine-3-HIB pathway on FA uptake and extracellular metabolites according to the metabolic context (e.g., with or without addition of FAs). Together, these data underscore a major impact of HIBCH perturbations on key metabolic processes in hepatocytes, which may represent mechanisms of altered metabolic flexibility in the pathogenesis of fatty liver disease.

Development of fatty liver and NAFLD largely results from a diet-induced increase in hepatic uptake and/or synthesis of lipids, without a sufficient compensatory increase in FA oxidation upon the increased hepatic FA load.^25^^,^^49^ Consistent with these previous observations, induction of lipid storage by adding FAs to our cultured hepatocytes upregulated genes that support oxidative phosphorylation, including *PDK4* which is a sensitive marker of FA oxidation.^28^ However, this increased mitochondrial respiration in our experiments was not a sufficient compensation to prevent marked lipid storage, and other anabolic genes must have facilitated the net intracellular lipid storage. Several of our observations indicate that elevated HIBCH plays a role in the mechanisms of lipid storage in hepatocytes. Firstly, *HIBCH* mRNA showed positive correlations with measures of fatty liver and adiposity and increased substantially in the hepatocytes that were induced to store large amounts of lipids. Secondly, HIBCH knockdown and overexpression in cultured hepatocytes reciprocally affected mitochondrial respiration, evidenced by altered expression of key genes that regulate FA oxidation and oxidative phosphorylation (e.g., *PDK4*, *CPT1A*, *ACOX2*, *PPARA*) as well as by increased oxygen consumption rate in cells with HIBCH knockdown. These data indicate that a lower HIBCH expression is permissive for greater cellular respiration, and conversely that higher HIBCH may prevent a sufficient compensatory increase in respiration upon nutritional challenge.

The marked effect we observed of *HIBCH* perturbations on PDK4 expression and a reciprocal effect of PDK4 inhibition on *HIBCH* expression, along with the increase in phosphorylated PDC upon HIBCH knockdown, moreover point to a role for HIBCH in the regulation of metabolic flexibility. Metabolic flexibility refers to the ability of a cell/organism to effectively switch between utilizing glucose or FAs, and may be critical to prevent excess hepatic lipid storage and fatty liver disease.^29^ Normally, PDC is active in the fed state, favoring glucose utilization over oxidation of FAs, while in a fasting state PDK4 inhibits PDC activity to favor oxidation of FAs.^27^ Importantly, inactivation of the PDC by overexpression of PDK4 is observed under diabetic conditions, and loss of regulatory flexibility of PDC is seen in NAFLD and other diseases.^27^^,^^29^ Consistent with our cohort data, elevated hepatic *PDK4* expression has previously been observed in patients with NAFLD/NASH.^46^ However, it should also be noted that mice with Pdk4 deficiency show reduced lipogenesis and hepatic steatosis in association with a marked downregulation of genes that regulate FA uptake and synthesis as well as gluconeogenesis.^46^ It remains to be determined if the upregulation of PDK4 we observed upon HIBCH knockdown reflects a protective feedback mechanism, or if the increased PDK4 expression contributed to perturbed metabolic flexibility in the hepatocytes. Notably, inborn HIBCH deficiency has been linked to reduced PDC activity and lower expression of enzymes in the respiratory chain in skeletal muscle and fibroblasts,^50^ supporting an important functional relationship between HIBCH and the PDK4/PDC mechanism.

Net hepatic lipid accumulation is a result of increased lipid uptake and synthesis and/or decreased oxidation. Previous studies applying HIBCH knockdown showed decreased lipid accumulation in white and brown adipocytes^20^ and decreased TAG synthesis and lipid droplet formation in piglet intestinal epithelial cells.^51^ Interestingly, in cultured hepatocytes we found that FA uptake increased modestly upon both knockdown and overexpression of HIBCH as well as upon PDK4 inhibition. This might be explained by distinct changes in the overall metabolic status induced by the respective perturbations (e.g., shift towards a more fasted or fed state), where incoming FAs serve different cellular purposes (FA storage or oxidation). Along the same reasoning, differences in FA oxidation induced by increased FA availability might explain why HIBCH knockdown showed a greater impact on several of the measured parameters in cells that were not treated with FAs, while overexpression more strongly affected FA-treated cells. Generally, the effects of HIBCH knockdown were abolished or counteracted by the FA addition. On the other hand, 3-HIB addition only affected FA uptake in the FA-treated hepatocytes, giving a significant decrease in FA uptake concomitant with a strong feedback downregulation of *HIBCH* mRNA. Taken together, the observed effects may reflect specific cellular adaptations required under different metabolic states, and suggest an important impact of altered HIBCH levels on hepatic energy- and lipid metabolism with implications for metabolic flexibility.

Metabolic modelling pointed to changes in the carnitine shuttle, and specifically lowered release of 2,6-dimethylheptanoyl (2,6-DML) carnitine from hepatocytes. The carnitine shuttle is essential for the transport of fatty acids into mitochondria for β-oxidation. The branched-chain fatty acyl 2,6-DML-CoA is a specific substrate of long-chain acyl-CoA dehydrogenase (LCAD), and a metabolic product of pristanic acid which in turn is formed by α-oxidation of phytanic acid.^52^ Notably, phytanic acid is exclusively derived from the diet, e.g., meat and dairy which are rich sources of BCAAs. Phytanic and pristanic acid are ligands for PPARα,^53^ a key transcriptional regulator of hepatic fatty acid β-oxidation. Thus, the decreased release of 2,6-DML carnitine upon loss of HIBCH may reflect impaired metabolism not only of valine but also of these diet-derived PPARα ligands.

ROS levels increase in conditions of excess hepatic lipid accumulation and are implicated by various mechanisms in the pathogenesis of fatty liver disease.^54^ HIBCH knockdown and 3-HIB addition showed consistent opposite effects on hepatic ROS levels, indicating that HIBCH and 3-HIB stimulate ROS generation. Notably, ROS levels decreased with the knockdown even though mitochondrial respiration increased, consistent with an increased capacity for ROS scavenging, which might potentially protect against the development of fatty liver disease.^54^ Increasing ROS generation and oxidative stress during hepatic lipid storage contribute to NAFLD, liver damage and fibrosis, primarily involving an increased pressure to dispose of lipids via FA β-oxidation in mitochondria and peroxisomes as well as cytochrome ω-oxidation and electron flux through the electron transport chain.^25^^,^^55^^,^^56^ Thus, increased FA oxidation without sufficient compensatory ROS scavenging makes hepatocytes vulnerable to oxidative stress, NAFLD and NASH, and further studies should explore whether targeted lowering of HIBCH activity in hepatocytes might be an effective therapeutic strategy to strengthen the capacity for both FA β-oxidation and ROS scavenging.

We also found increased AKT phosphorylation upon HIBCH knockdown, suggesting that HIBCH may contribute to hepatic insulin resistance, in line with a reduced insulin response upon 3-HIB exposure of myocytes^21^^,^^45^ and adipocytes.^20^ This permissive effect of HIBCH loss on AKT phosphorylation in hepatocytes was likely not mediated via the increased PDK4 expression in cells with HIBCH knockdown, since inhibition of PDK4 was found previously to enhance hepatic insulin/Akt signalling and activate a pathway that regulates glucose- and lipid metabolism (as well as promotes liver regeneration), involving AMPK, FOXO1 and CD36.^57^ Interestingly, gene expression in the cultured hepatocytes with HIBCH overexpression or knockdown pointed to HIBCH-mediated suppression of the fetal growth factor member FGF18 and the FGF receptors 1–3, as well as IRS2. IRS2 is essential for insulin signalling in hepatocytes,^58^ and is also suppressed by the key lipogenic transcription factor SREBP.^59^ Conversely, our data showed that HIBCH knockdown lowered and HIBCH overexpression tended to increase expression of *PIK3CA* (encoding the p110α subunit of PI3K) and *GRB10*. GRB10 is stabilized by the BCAA-responsive mammalian target of rapamycin (mTOR, a kinase partly activated by Akt), which allows GRB10 to suppress insulin signalling through feedback inhibition of PI3K.^60^ More detailed studies should explore the mechanisms for how HIBCH and its product 3-HIB may contribute to hepatic lipid accumulation by modulating specific parts of the insulin signalling cascades and related feedback systems.

Exploring HIBCH as a therapeutic target is further motivated by the markedly increased extracellular MMA concentrations we observed upon HIBCH overexpression, as MMA (an established marker of vitamin B12 status) has been identified as a marker of advanced liver fibrosis in patients with NAFLD.^61^ The link between HIBCH and MMA is further supported by a previous study showing higher plasma MMA concentrations in people carrying a missense mutation in HIBCH,^62^ although it remains to be determined in which tissue(s) this genetic variant acts. Additionally, in male Wistar rats, we previously found that increased mitochondrial FA oxidation by a synthetic FA analogue upregulated hepatic *Hibch* mRNA 3-fold concomitant with a 3-fold increase in plasma MMA.^22^ While these increases are consistent with a positive relationship between hepatic *HIBCH* expression and circulating MMA, the increased *Hibch* mRNA and MMA were seen in the context of a strongly stimulated FA oxidation, a key property of the synthetic FA analogue used.^63^ It should be noted that these data were correlative as opposed to our direct perturbations of hepatic HIBCH in the present study and that the causal relationships in different metabolic contexts need to be further investigated. Nevertheless, the different studies clearly demonstrate a relationship between HIBCH and circulating MMA levels, and the present study indicates that the liver is an important player in this interaction.

The present study has limitations. First, although the Huh7 immortalized human cell line serves as an *in vitro* model for studying fatty liver,^32^ this is a male-derived hepatoma line with some unique metabolic properties compared to other models such as HepG2 hepatoma cells and primary hepatocytes.^33^^,^^43^ While we confirmed key results across the Huh7 cells and liver biopsies, replication studies in even more models including primary cells from both males and females are needed. Second, we could not directly relate plasma 3-HIB concentrations to *HIBCH* expression in liver biopsies, since both expression and metabolite data were not available in the same cohorts. Third, we used culture medium relatively high in glucose in combination with increased FA supply, and other effects may have been observed in low-glucose conditions that are more conducive to FA oxidation. Nonetheless, marked increases in FA β-oxidation are observed with PDK4 overexpression also in cells supplied with 25 mM glucose.^28^ Fourth, our experimental studies were based on cell cultures treated for 24 h, and we cannot necessarily extrapolate the findings to more long-term treatment or the *in vivo* regulation of metabolic homeostasis. Fifth, future studies of effects of HIBCH and 3-HIB on hepatic lipid accumulation should more comprehensively measure protein levels and enzyme activities, such as of lipogenic enzymes. Finally, HIBCH not only generates 3-HIB but also 3-hydroxypropionate, and the metabolic impact of altered HIBCH expression via these respective steps needs clarification.

In conclusion, our data indicate that HIBCH facilitates lipid storage in hepatocytes, in part by suppressing mitochondrial FA oxidation via inhibition of PDK4. Upon nutritional challenge over time, increased HIBCH activity promote excess lipid storage and may manifest in reduced metabolic flexibility.

## Contributors

M.S.B. and S.N.D. designed the study, researched data and wrote the manuscript. M.K., M.B., G.M., C.H., J.L-B. and S.N.D. designed and organized the clinical cohort studies. M.S.B. carried out the experiments. L.L-A., M.S.B and S.N.D. analyzed the RNA sequencing data. A. Mc. and J.L-B. contributed to metabolite analyses. R.C.G. and I.G.J. performed the metabolic modelling. M.S.B., L.L-A. and S.N.D. analyzed and interpreted the results. All authors reviewed and approved the final version of the manuscript. M.S.B and S.N.D. are the guarantors of this work and, as such, had full access to all the data in the study and takes responsibility for the integrity of the data and the accuracy of the data analysis.

## Data sharing statement

All data are available in the main text, the supplementary materials or deposited in a public database (see methods section: “Data deposition”).

## Declaration of interests

S.N.D. received grants to the institution (University of Bergen) from the Research Council of Norway, the Novo Nordisk Scandinavia AS and the Norwegian Diabetes Association. M.B. has received personal honoraria for consultations/presentations from Amgen, AstraZeneca, Bayer, Boehringer-Ingelheim, Lilly, Novo Nordisk, Novartis and Sanofi, and served on an advisory board for Boehringer-Ingelheim. G.M. received funding from the Trond Mohn Foundation (to the University of Bergen), consulting fees from Novo Nordisk Norway and payment for expert testimony from the Throne Holst Nutrition Research Foundation, Norway.

## Appendix A Supplementary data

Supplemental western blots
