# Supplementary figures and images for: Metabolic role of the hepatic valine/3-hydroxyisobutyrate (3-HIB) pathway in fatty liver disease
Source: eBioMedicine. 2023 Apr 19;91:104569. doi: 10.1016/j.ebiom.2023.104569 (PMC10148099; doi:10.1016/j.ebiom.2023.104569)

Full unedited blots

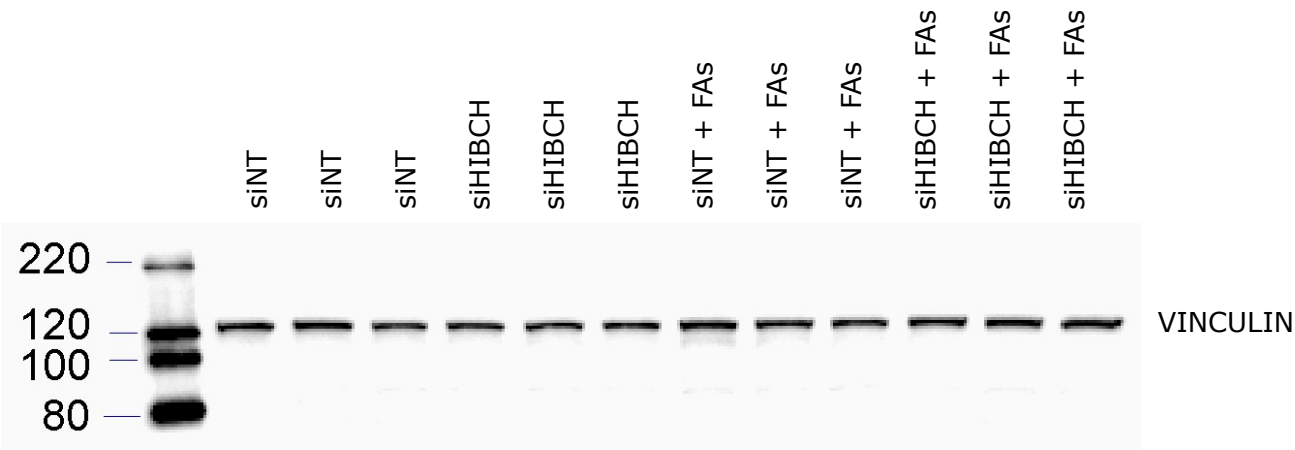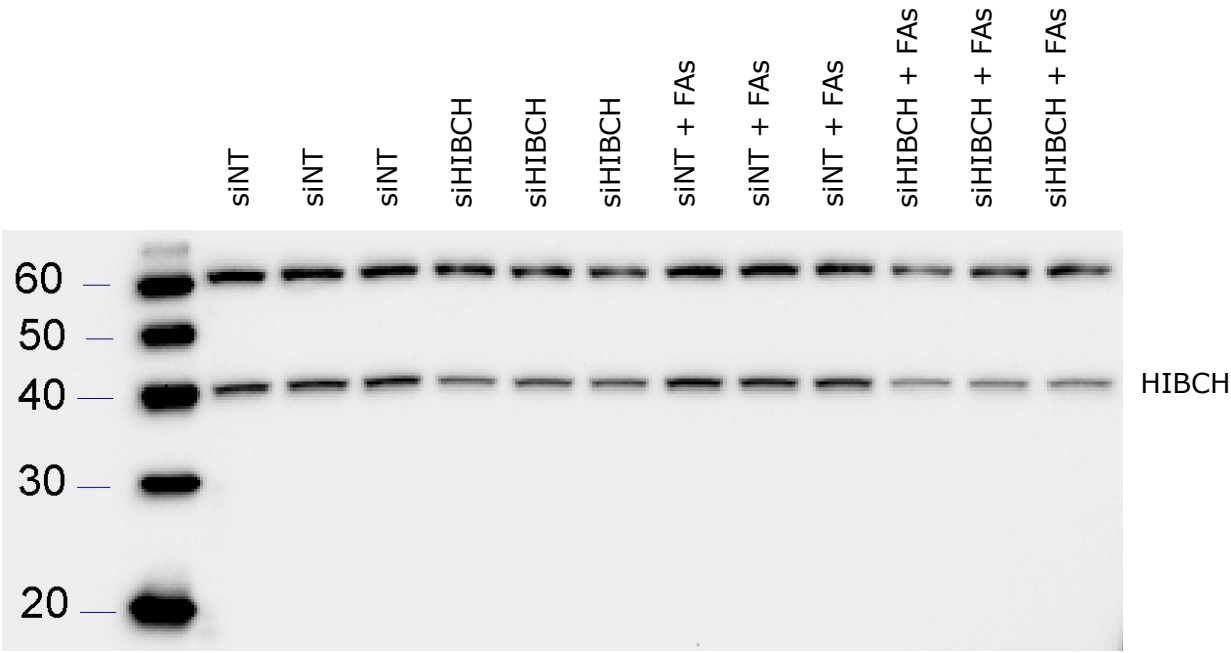

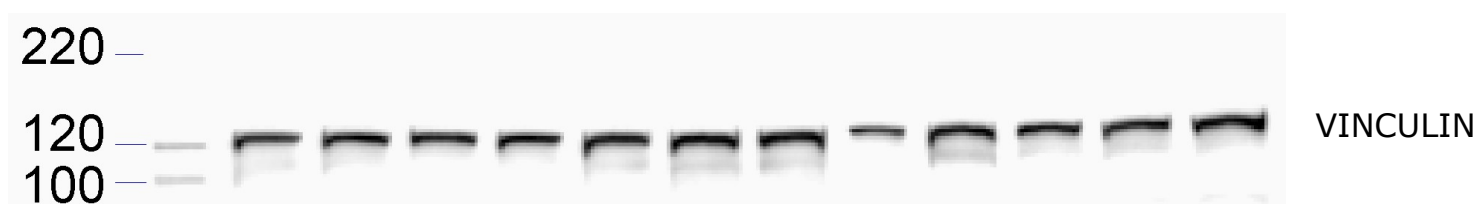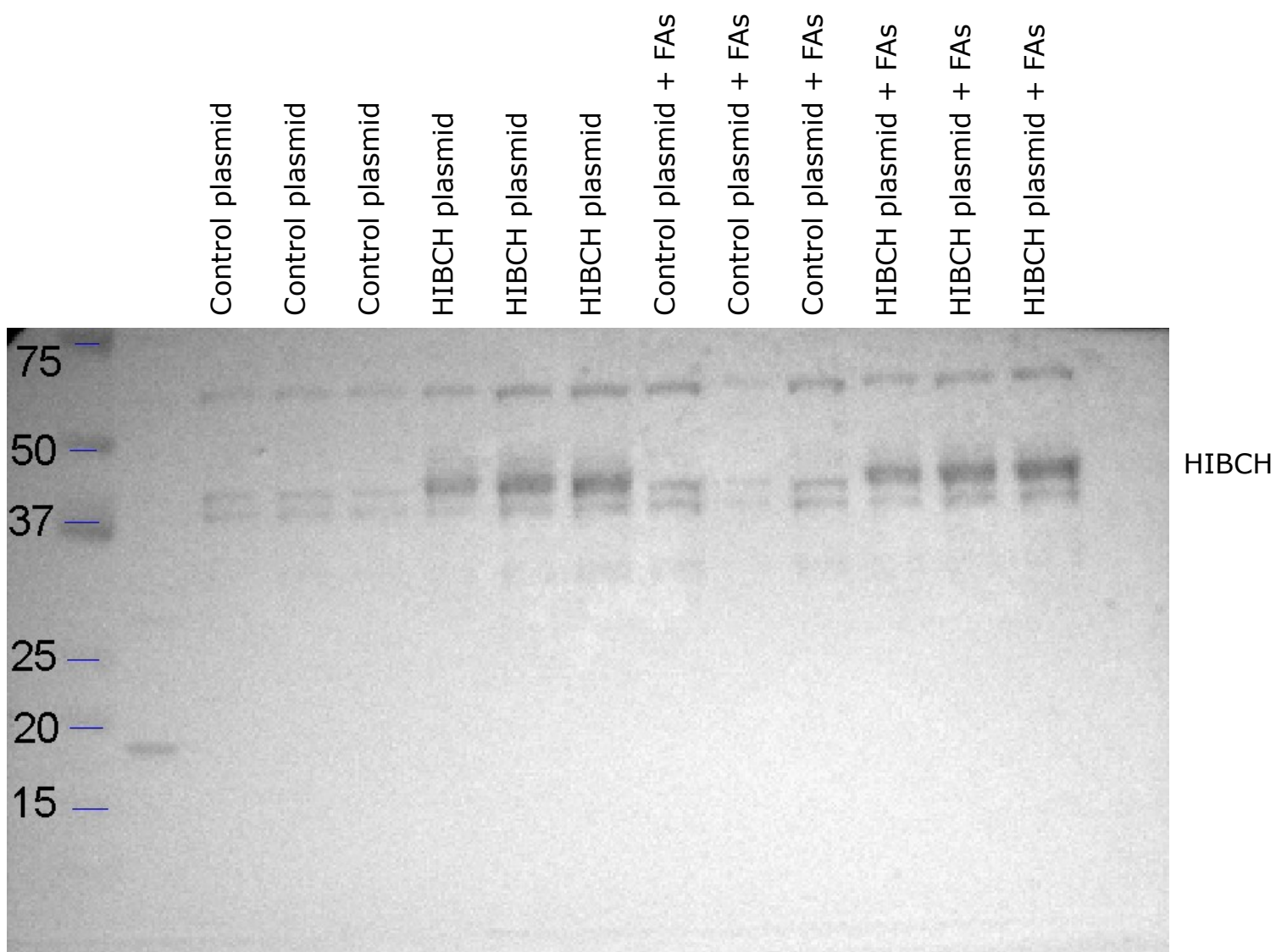

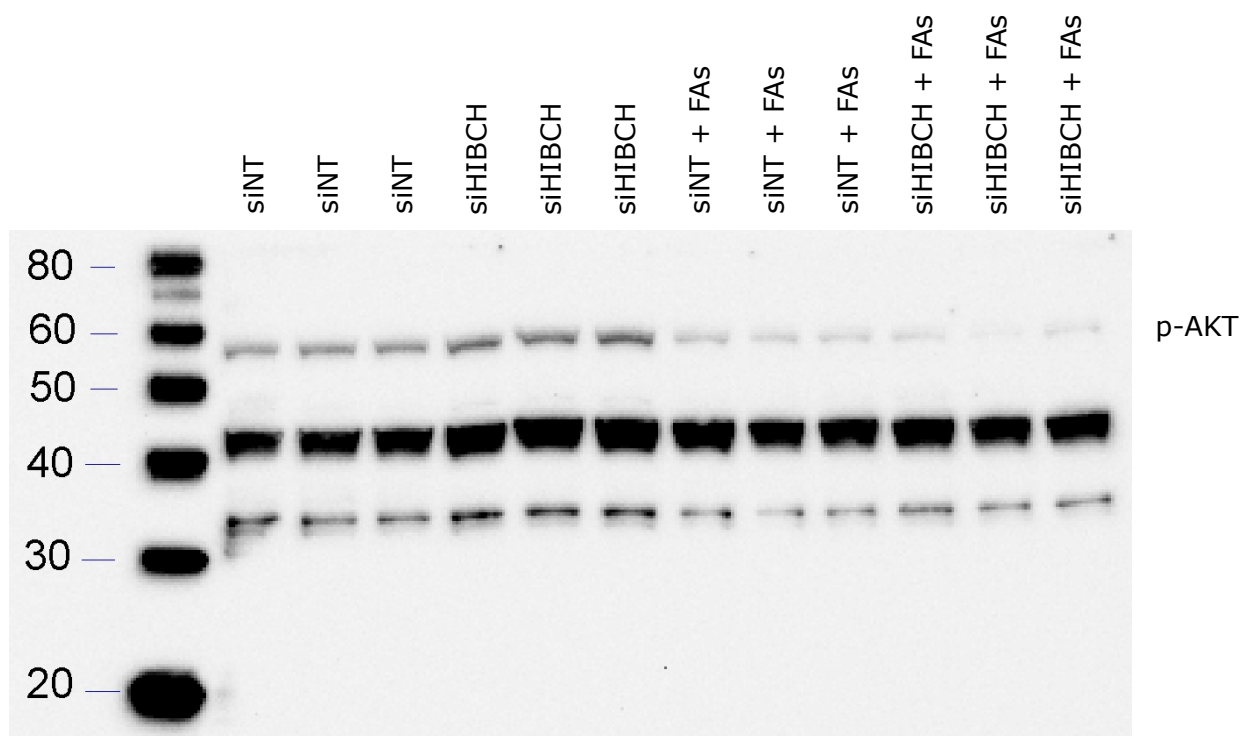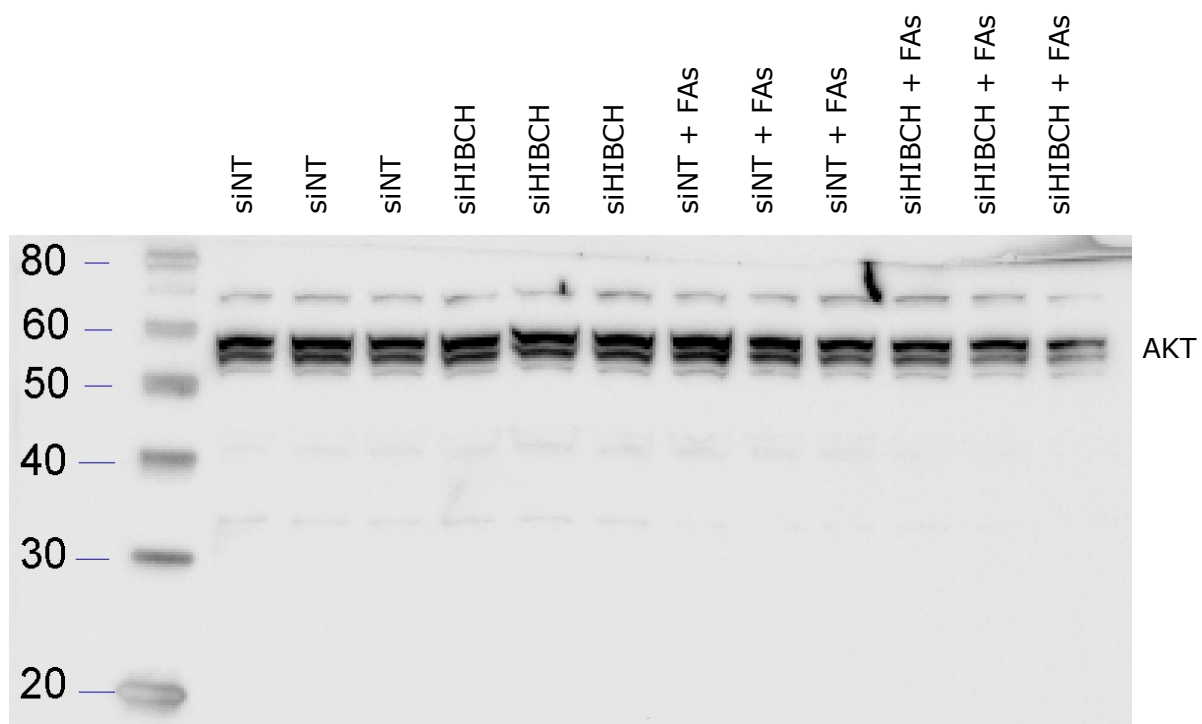

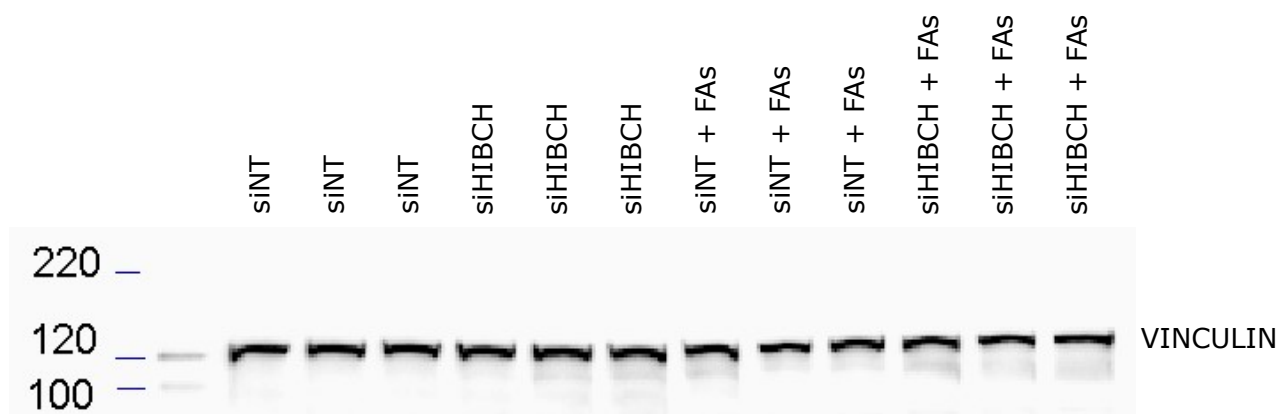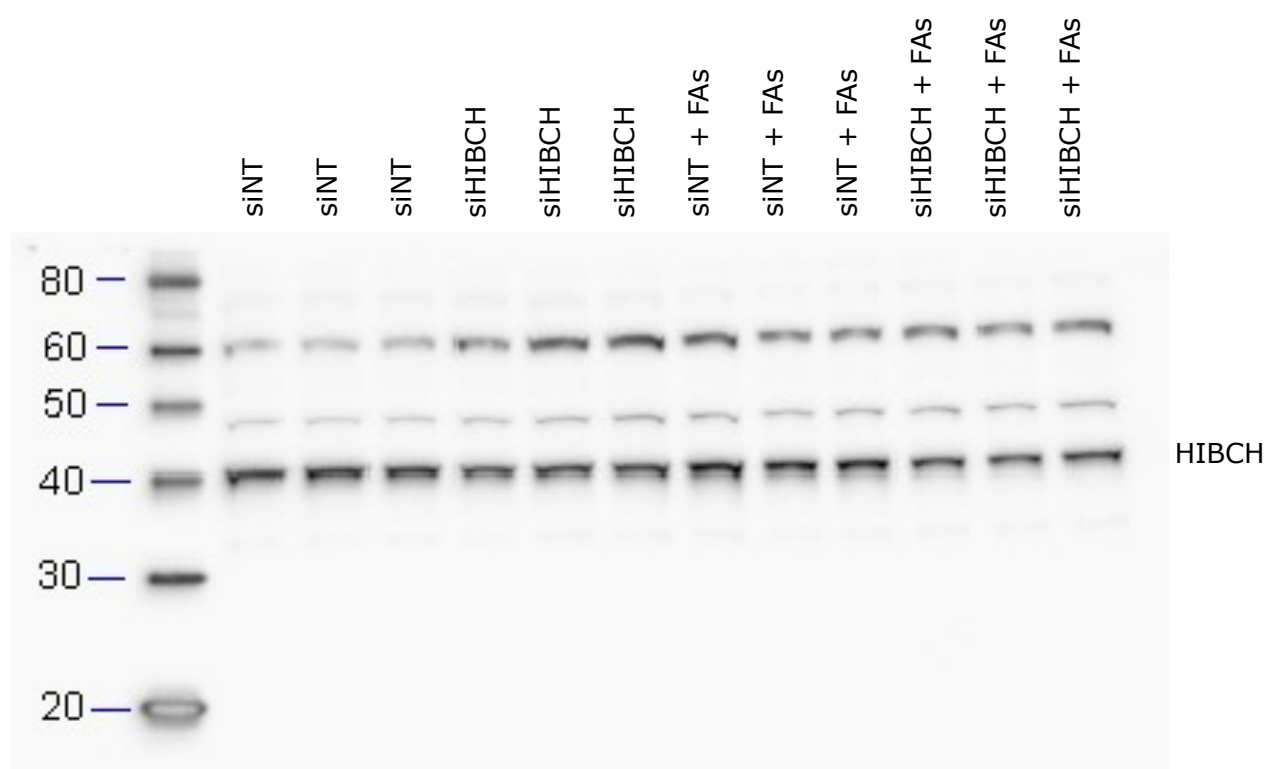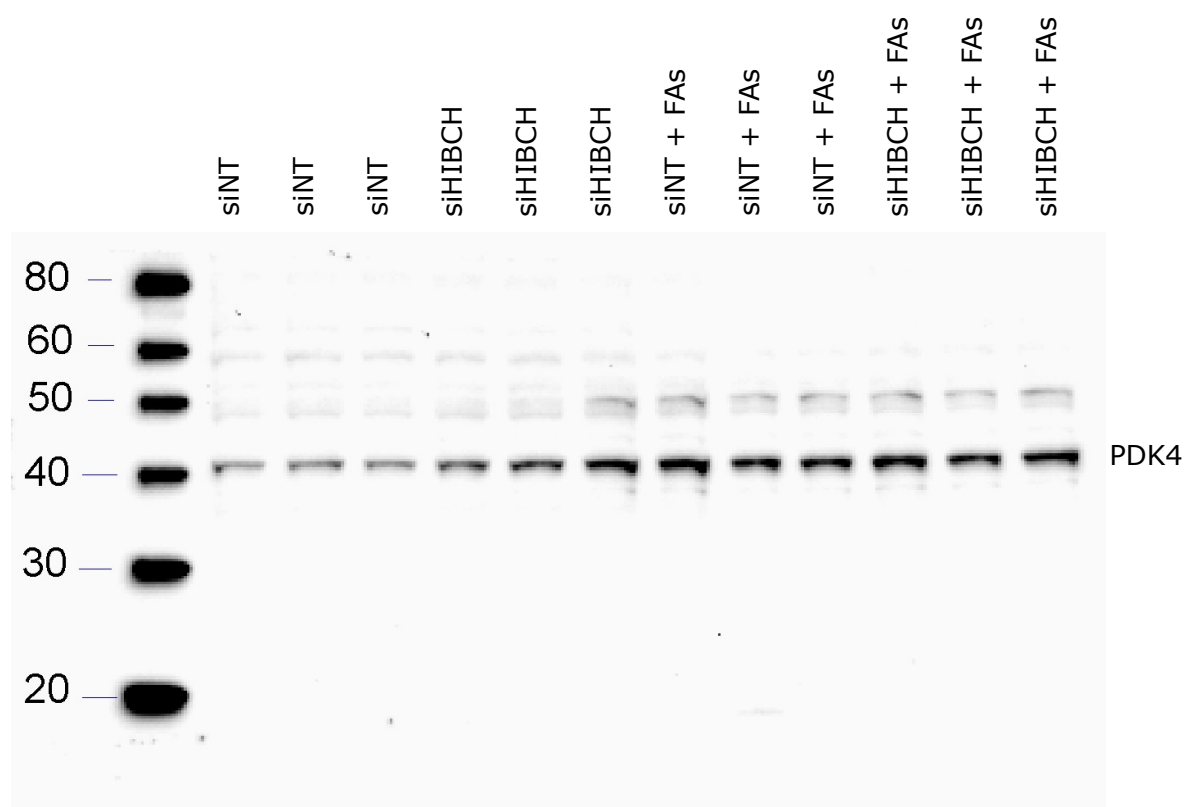

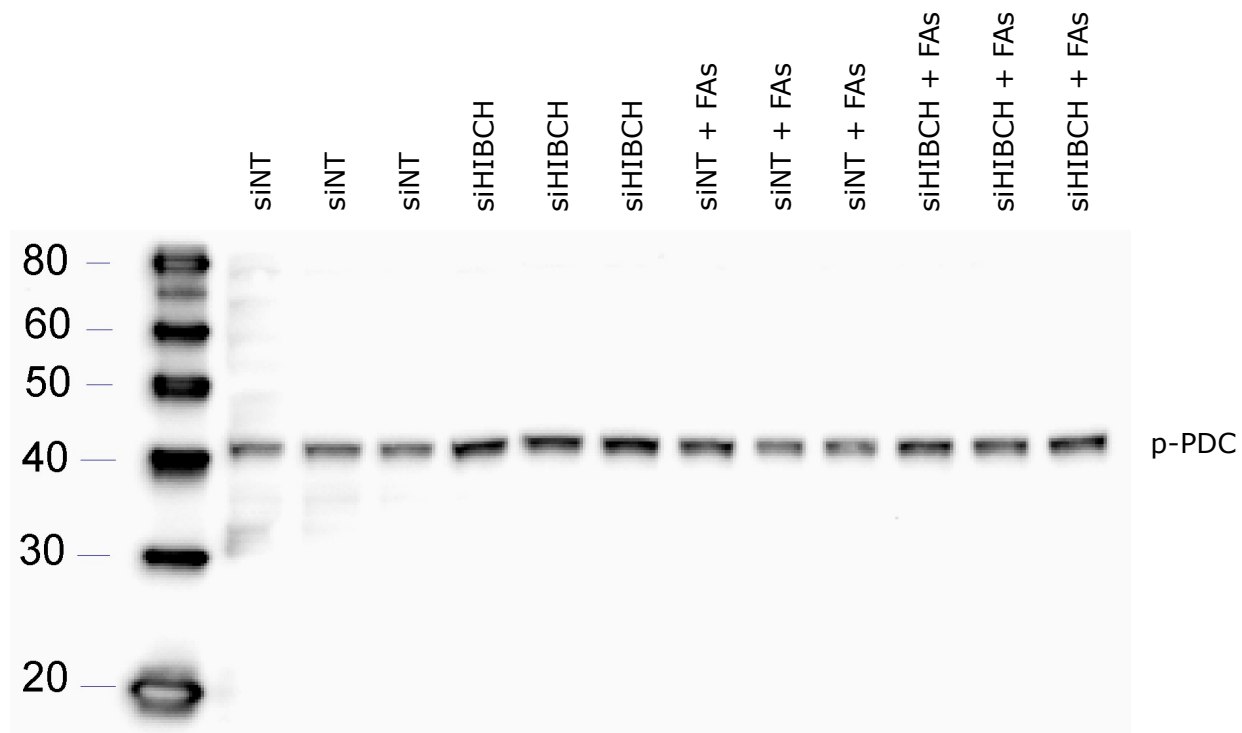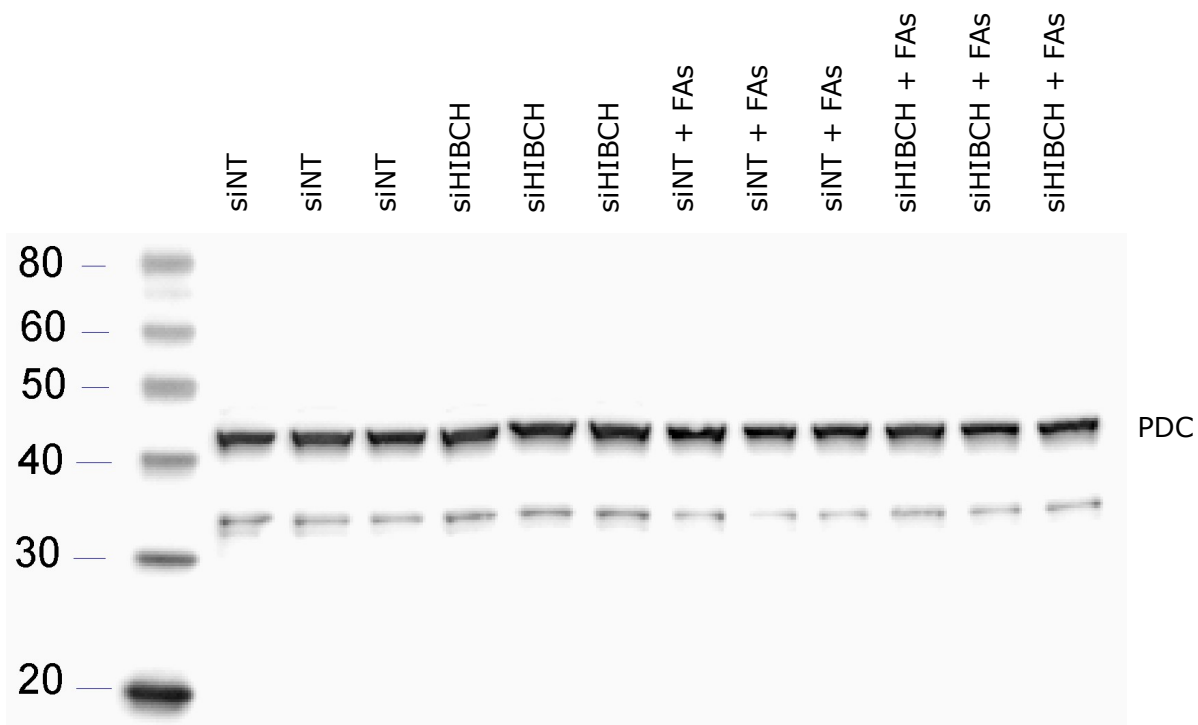

Supplement: Supplemental western blots [file mmc1.pdf]
