# Supplementary material for: Metabolic role of the hepatic valine/3-hydroxyisobutyrate (3-HIB) pathway in fatty liver disease
Source: eBioMedicine. 2023 Apr 19;91:104569. doi: 10.1016/j.ebiom.2023.104569 (PMC10148099; doi:10.1016/j.ebiom.2023.104569)
Supplement: Supplementary material Tables S1–S3 and Figs. S1–S9 [file mmc2.pdf]

SUPPLEMENTARY MATERIALS

Bjune et al. Metabolic role of the hepatic valine/3-hydroxyisobutyrate (3-HIB) pathway in fatty liver disease. *eBioMedicine*.

Contents

Supplementary Figure 1. Correlations of valine with clinical features and metabolites.

Supplementary Figure 2. Effects of FA treatment on lipid accumulation and global gene expression in cultured human hepatocytes.

Supplementary Figure 3. Pathways and metabolic functions affected by FA treatment of cultured human hepatocytes.

Supplementary Figure 4. Effects of HIBCH overexpression on the transcriptome in cultured human hepatocytes.

Supplementary Figure 5. Effects of HIBCH knockdown on the transcriptome in cultured human hepatocytes.

Supplementary Figure 6. Biological processes affected by HIBCH knockdown in cultured human hepatocytes.

Supplementary Figure 7. HIBCH-dependent regulation of insulin signaling in cultured human hepatocytes.

Supplementary Figure 8. HIBCH knockdown upregulates PDK4 protein expression and mitochondrial respiration and TCA cycle metabolites in cultured human hepatocytes.

Supplementary Figure 9. Mitochondrial respiration following 3-HIB supplementation in cultured human hepatocytes.

Supplementary Table 1. Clinical characteristics of participants in the studied cohorts.

Supplementary Table 2. Spearman correlations (adjusted for age and sex) for plasma metabolites and liver fat or BMI in CARBFUNC cohort.

Supplementary Table 3. All reactions affected by HIBCH knockdown in constraint-based metabolic modeling of human liver cells (iHepatocytes2322).

Supplementary Figures

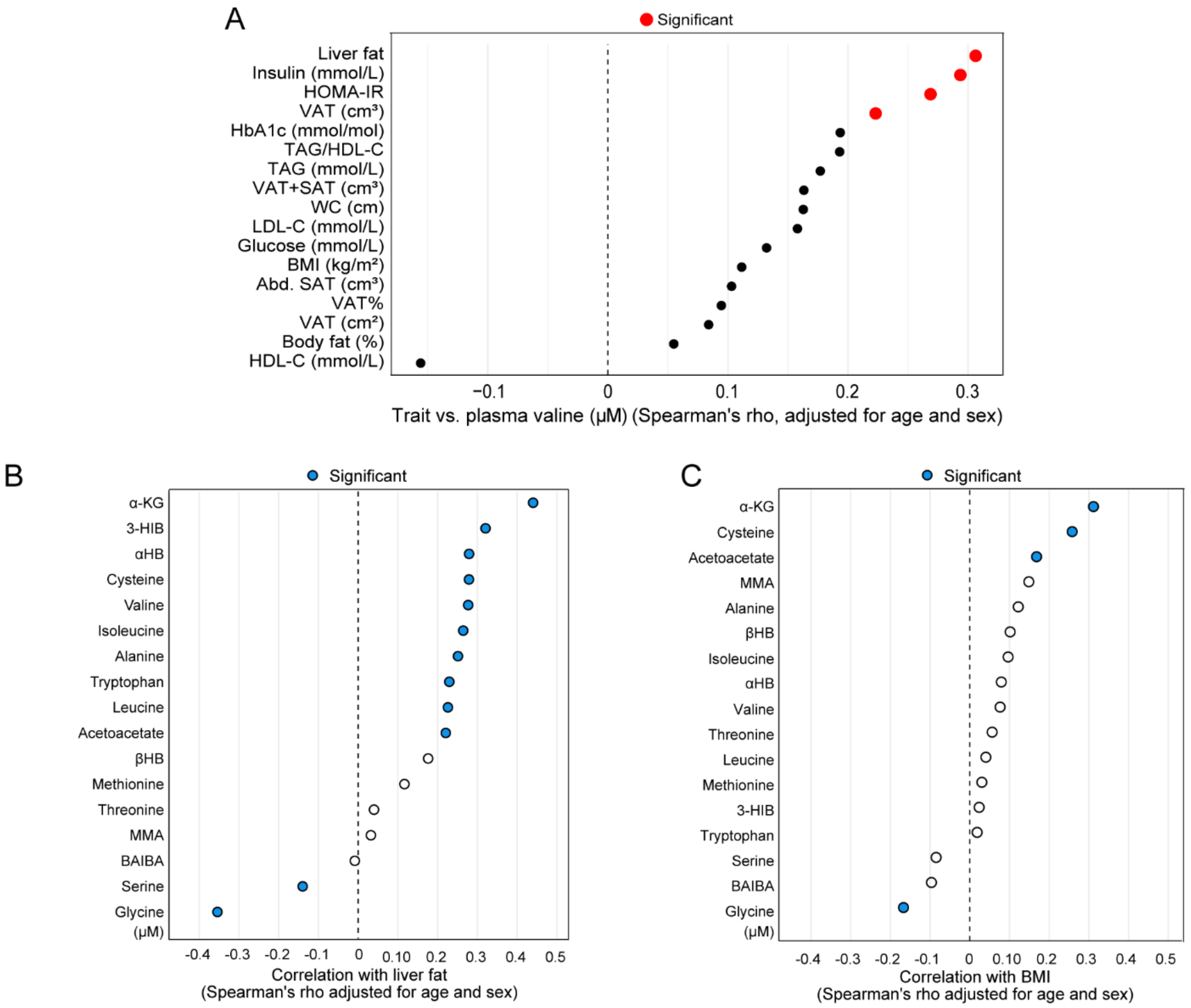

Supplementary Figure 1. Correlations of valine with clinical features and metabolites. A: Graphical representation of Spearman correlations for plasma valine and different variables in 192 individuals with abdominal obesity (BMI ≥ 30 kg/m^2) and/or WC ≥ 102 cm (for men) and WC ≥ 88 cm (for women) (CARBFUNC cohort). Liver density was measured by CT imaging and calculated as HU units and divided by spleen density. Because increased liver density reflects lower fat content, the correlation coefficient in the figure was inverted to a positive value (i.e., reflecting more liver fat). B-C: Graphical representation of Spearman correlations for measured metabolites/amino acids and liver fat and BMI in CARBFUNC.

SAT, subcutaneous adipose tissue; TG, triglycerides; VAT, visceral adipose tissue; WHR; Waist-to-Hip-Ratio.

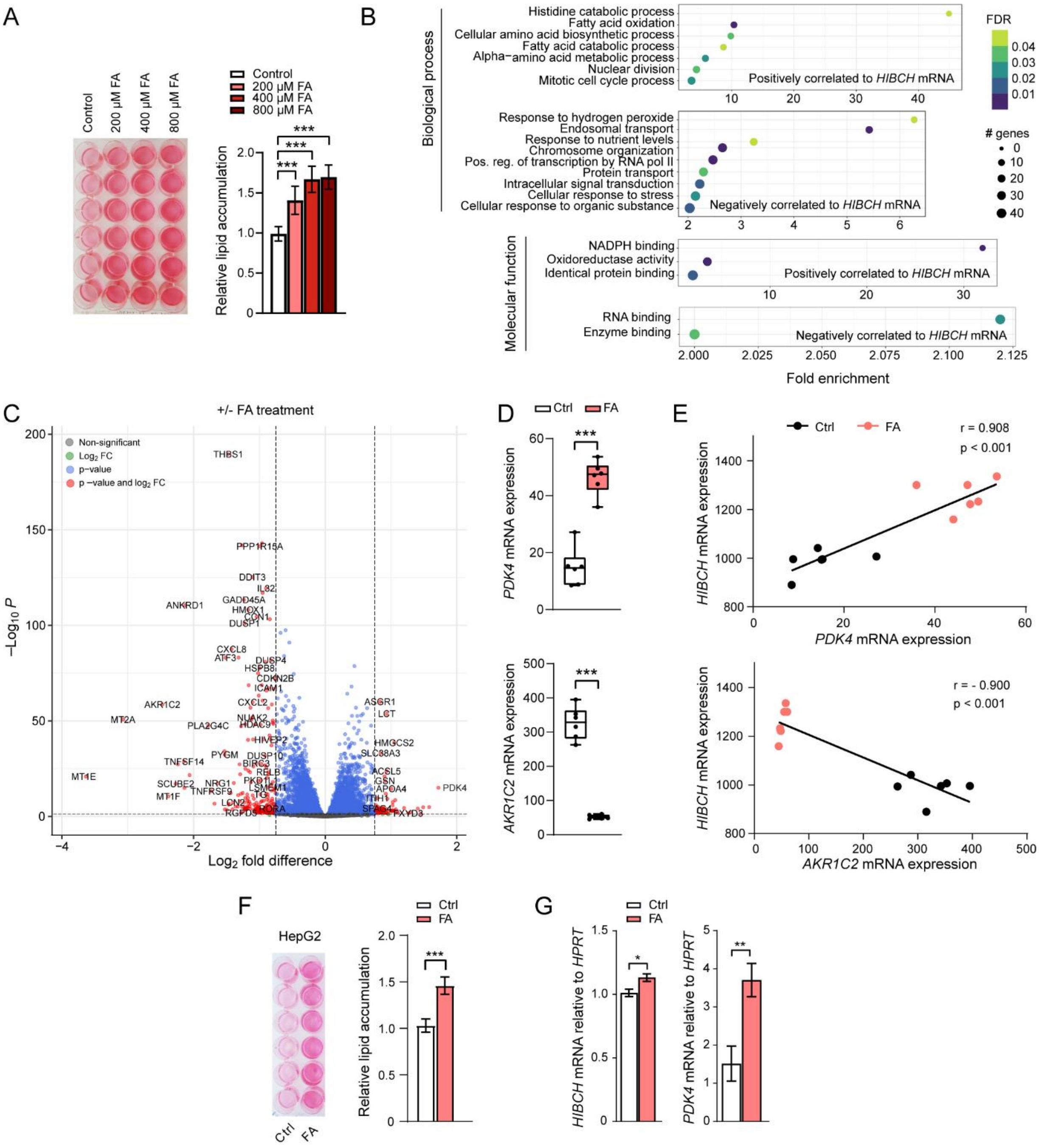

Supplementary Figure 2. Effects of FA treatment on lipid accumulation and gene expression in cultured human hepatocytes. Huh7 and HepG2 liver cells were treated with and without free FAs (1:1 molar ratio of 50 µM PA and 50 µM OA, unless otherwise specified) for 24h before analyses. A: Picture of wells containing Oil-Red-O lipid-stained Huh7 hepatocytes (left) and quantification of lipid accumulation (right) in control and cells treated with different doses of free FAs, PA and OA (1:1 molar ratio) for 24h. The total FA treatment doses are presented in the figure (n = 6). B: HIBCH correlation of RNA sequencing data set was used to perform Panther GO analyses (biological process and molecular function) showing pathways that are positively and negatively correlated to hepatic *HIBCH* expression in Huh7. Up-and down-regulated pathways are shown with fold enrichment visualized on the x-axis and the number of genes included in each pathway is shown by the size of the circle. The color of the circle indicted level of statistical significance shown by false discovery rate (FDR) (the results of the analyses with FDR < 0.05 are shown). C: Volcano-plot of RNA sequencing data showing genes that are up- and downregulated by FA treatment in Huh7. D: *PDK4* and *AKR1C2* expression (RPKM) in Huh7 cells (n = 6). E: *HIBCH* mRNA expression correlated to *PDK4* and *AKR1C2* expression (RPKM) in Huh7 cells (n = 6) F: Picture of wells containing Oil-Red-O lipid-stained HepG2 hepatocytes (left) and quantification of lipid

accumulation (right) (n = 6). G: *HIBCH* and *PDK4* expression in HepG2 cells measured by qPCR, calculated relative to the reference gene *HPRT* (n = 3).
Ctrl, control; FA, fatty acid treatment; FDR, false discovery rate.
*P < 0.05, **P < 0.01, ***P < 0.001 (Ordinary one-way ANOVA - Sidak's test).

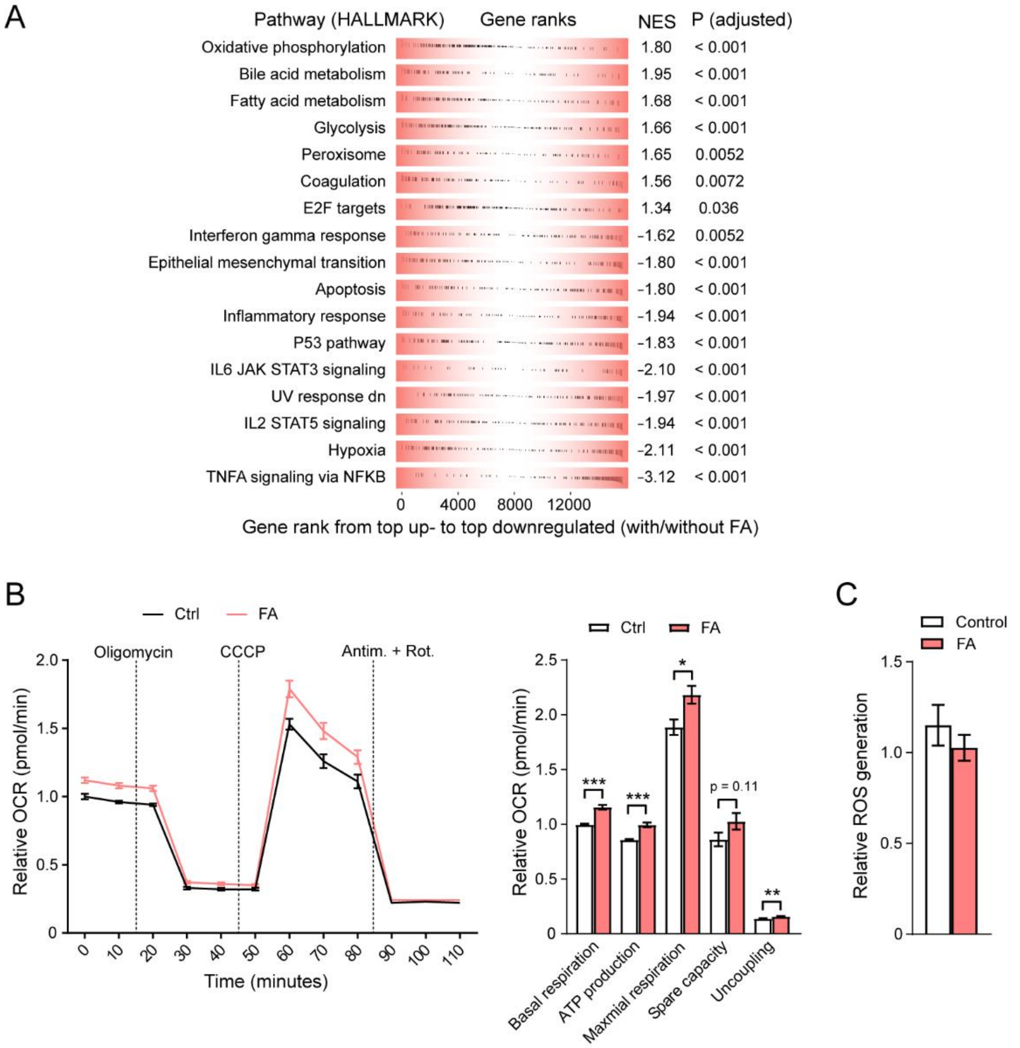

Supplementary Figure 3. Pathways and metabolic functions affected by FA treatment of cultured human hepatocytes. Huh7 liver cells were treated with and without free FAs (1:1 molar ratio of 50 µM PA and 50 µM OA) for 24h before analyses. Gene expression was measured by RNA-sequencing. A: Gene Set Enrichment analysis (GSEA) showing up- and downregulated pathways by FA treatment in Huh7 cells (HALLMARK pathway analysis of RNA sequencing data) (n = 6). Gene sets are ordered by normalized enrichment score (NES) and significant p-value and adjusted p-value for each pathway are shown. B: Seahorse Cell Mito Stress Assay (OCR measurements) was performed using the Seahorse XFe96 Analyzer to assess the mitochondrial respiration in Huh7 (n = 10-12) 24h after FA treatment. Basal levels (the three first OCR measurements) were obtained, before adding oligomycin, CCCP and rotenone/antimycin A, as indicated at the top in the upper left figure. Outliers were removed based on a Whisker Tukey test of the OCR data for each time point in each well. Basal respiration, ATP production, maximal respiration, spare capacity and uncoupling were calculated for each well based on the OCR measurements. C: Relative values of ROS production in Huh7 cells treated with or without FA for 24 hours, followed by addition of fluorescent probe whose uptake was detected spectrophotometrically after 1 hour (n = 11).
Antim. + Rot., antimycin + rotenone; adenosine triphosphate production; CCCP, carbonyl cyanide 3-chlorophenol hydrazone; FA, fatty acid; NES, normalized enrichment score.
*P < 0.05, **P < 0.01, ***P < 0.001 (unpaired t-test or MannWhitney test).

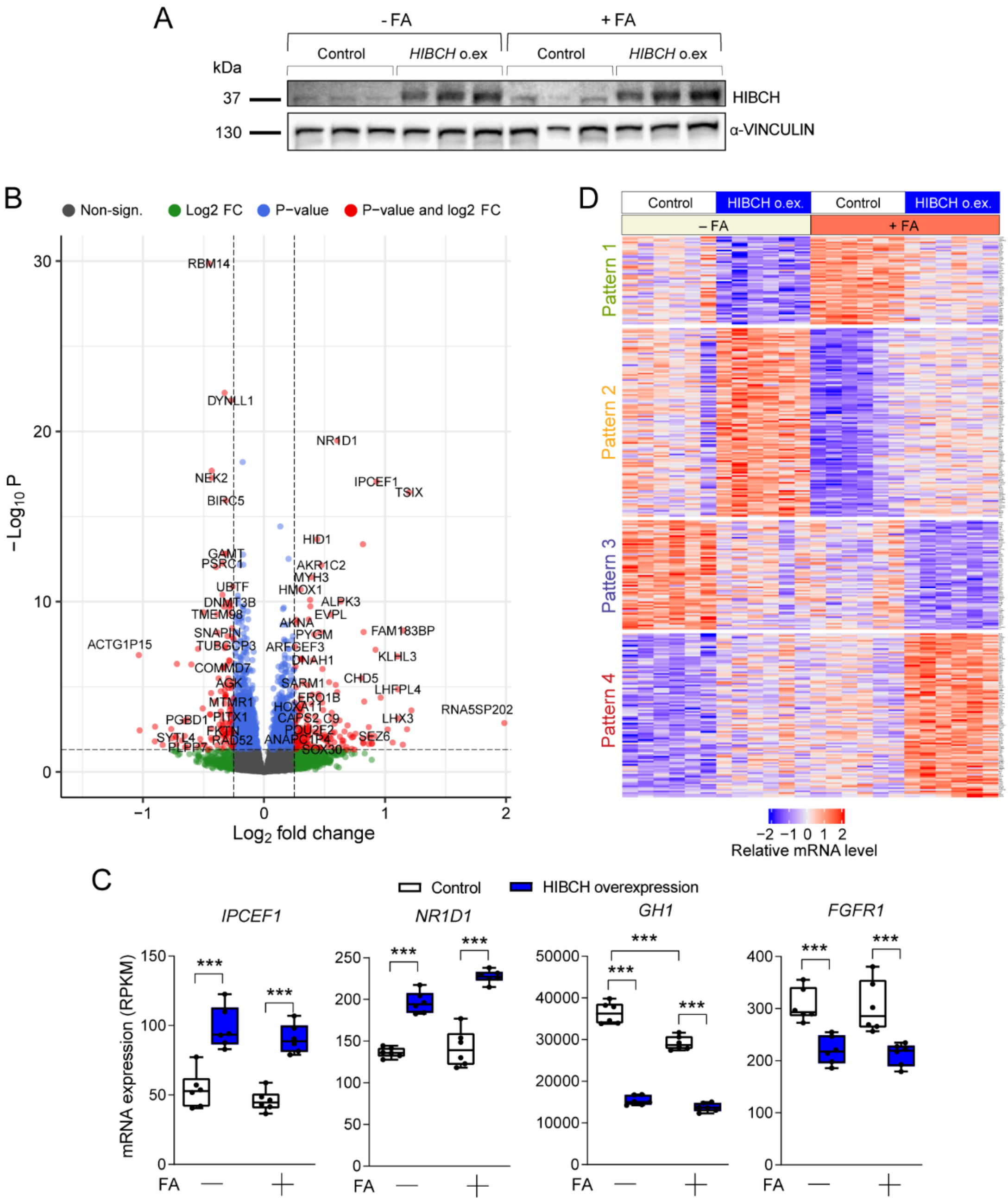

Supplementary Figure 4. Effects of HIBCH overexpression on the transcriptome in cultured human hepatocytes. Huh7 liver cells were transfected with pCMV6-HIBCH or control (pCMV6-empty vector) plasmid (0.2 µg per well in a 24-well plate) diluted in Opti-MEM® Reduced Serum Media and TransIT-X2® transfection reagent (Mirus). 24h after transfection, the cells were treated with and without free FAs (1:1 molar ratio of 50 µM PA and 50 µM OA) for 24h before analyses. A: Western blot showing protein levels of HIBCH and α-VINCULIN (loading control) (n = 3). B: Volcano plot of differentially expressed genes between control and HIBCH overexpression (combining samples with and without FA treatment) (n = 24). X-axis corresponds to log2 fold change (log2FC) and y-axis corresponds to -log10 (p-value). Red dots represent the transcript with an absolute log2FC > 0.5 and p-value < 10E-10; green dots represent the transcripts with an absolute log2FC > 0.5; blue dots represent transcript with p-value < 10E-10; grey dots represent transcripts which do not pass above thresholds. C: Gene expression of *IPCEF1*, *NR1D1*, *GH1* and *FGFR1* (n = 6). D: RNA sequencing data shown as a heatmap comparing the effect of the four treatments (n = 6 per treatment) for genes in the four different gene expression patterns. Relative mRNA level is indicated as the intensity of blue (decreased expression) and red (increased expression) color.

FA, fatty acid treatment; HIBCH o.ex., HIBCH overexpression.

*P < 0.05, **P < 0.01, ***P < 0.001 (Ordinary one-way ANOVA - Sidak's test)

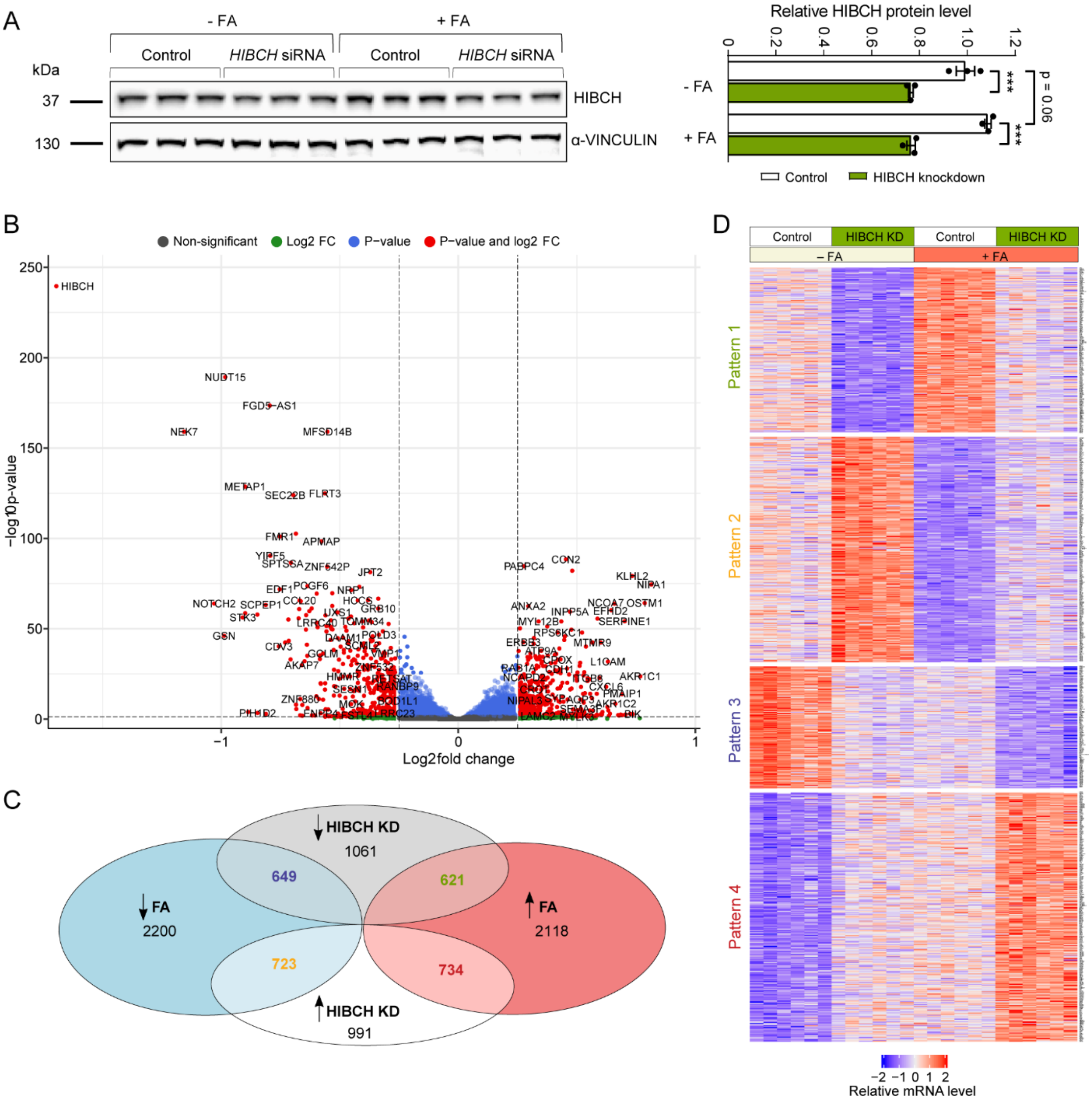

Supplementary Figure 5. Effects of HIBCH knockdown on the transcriptome in cultured human hepatocytes. Huh7 liver cells were treated with and without free FAs (1:1 molar ratio of 50 µM PA and 50 µM OA) combined with siRNA-mediated knockdown of HIBCH and siRNA non-targeting control for 24h before analyses. Gene expression was measured by RNA-sequencing. A: Western blot showing protein levels of HIBCH and α-VINCULIN (loading control) (left) and the quantitative values of HIBCH relative to α-vinculin (right) (n = 3). B: Volcano plot of differentially expressed genes between control and HIBCH knockdown (combining samples with and without FA treatment to focus on the knockdown effect) (n = 24). X-axis corresponds to log2 fold change (log2FC) and y-axis corresponds to -log10 (p-value). Red dots represent the transcript with an absolute log2FC > 0.5 and p-value < 10E-10; green dots represent the transcripts with an absolute log2FC > 0.5; blue dots represent transcript with p-value < 10E-10; grey dots represent transcripts which do not pass above thresholds. C: Number of up- and down-regulated genes by HIBCH knockdown with/without FA treatment in Huh7 cells shown as a Venn diagram (adjusted P < 0.1). D: RNA sequencing data shown as a heatmap comparing the effect of the four treatments (n = 6 per treatment) for genes in the four different gene expression patterns. Relative mRNA level is indicated as the intensity of blue (decreased expression) and red (increased expression) color. FA, fatty acid treatment; KD, knockdown.

*P < 0.05, **P < 0.01, ***P < 0.001 (Ordinary one-way ANOVA - Sidak's test).

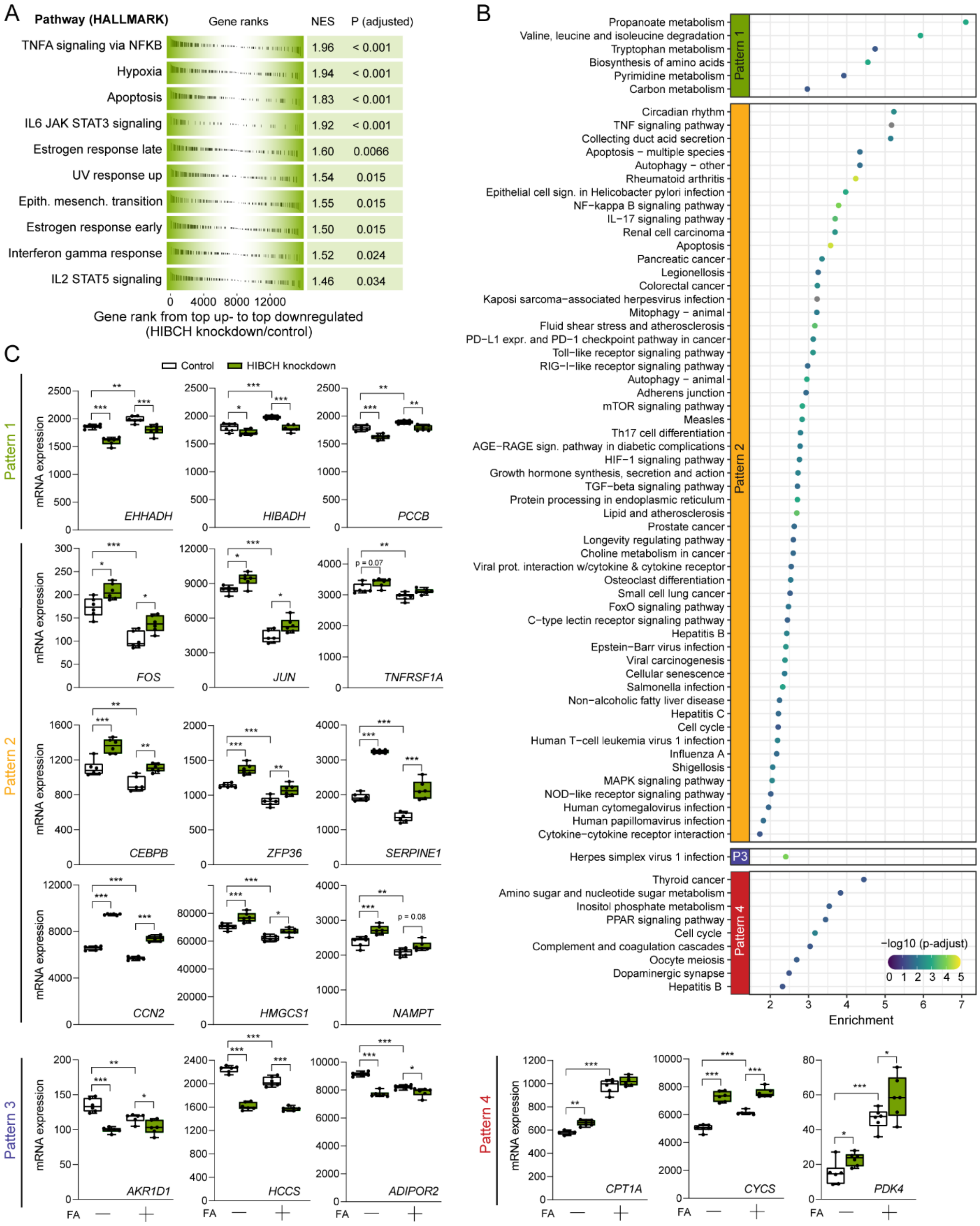

Supplementary Figure 6. Biological processes affected by HIBCH knockdown in cultured human hepatocytes. Gene expression was measured by RNA sequencing. A: GSEA showing up- and down-regulated pathways by HIBCH KD relative to control expression (without and with FAs) in Huh7 (HALLMARK pathway analysis) (n = 24). Gene sets are ordered by normalized enrichment score (NES) and significant adjusted p-value for each pathway are shown. B: KEGG pathway analysis of the four gene expression patterns. C: Gene expression

data in control and HIBCH knockdown Huh7 cells (without and with FA treatment) shown as RPKM (n = 6). FA, fatty acid treatment; GSEA, gene set enrichment analysis; KD, knockdown; RPKM, reads per kilobase per million mapped reads. FA, fatty acid treatment; NES, normalized enrichment score.
*P < 0.05, **P < 0.01, ***P < 0.001 (Ordinary one-way ANOVA - Sidak's test).

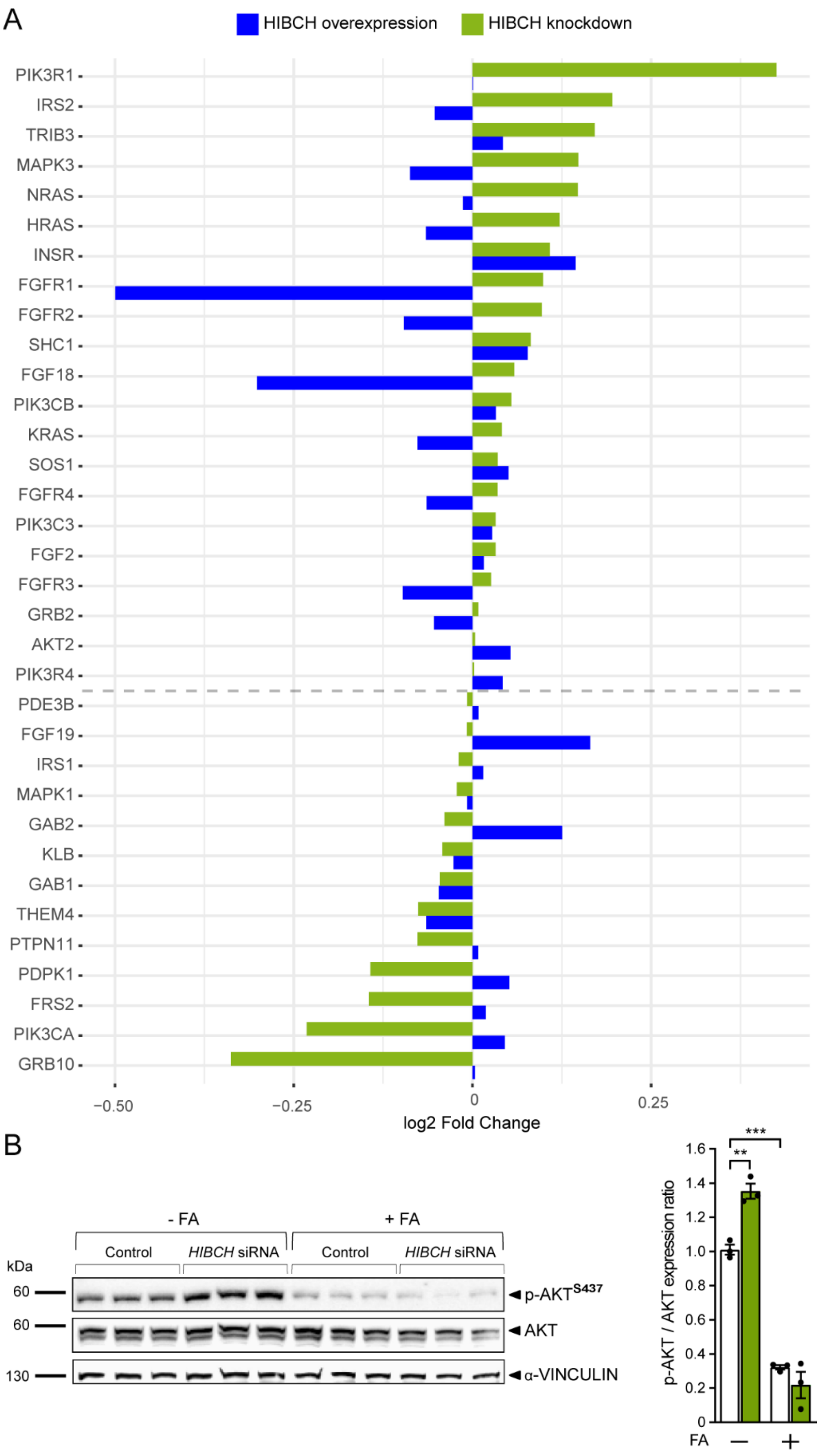

Supplementary Figure 7. HIBCH-dependent regulation of insulin signaling in cultured human hepatocytes. Huh7 liver cells were transfected with siRNA-mediated knockdown of HIBCH and siRNA non-targeting control or with pCMV6-HIBCH or control (pCMV6-empty vector) plasmid (0.2 µg per well in a 24-well plate) diluted in Opti-MEM® Reduced Serum Media and TransIT-X2® transfection reagent (Mirus). The cells were treated with and without free FAs (1:1 molar ratio of 50 µM PA and 50 µM OA) for 24h, before analyses. A: GSEA of RNA sequencing data showing up- and down-regulated genes (as log2 fold change) in the gene set "insulin receptor signalling cascade" by knockdown and overexpression of HIBCH in Huh7 cells (data for cells treated with and without FA combined). B: Western blots showing protein levels of p-AKT, AKT and α-VINCULIN (loading control) (left) and the quantitative values of p-AKT/AKT ratio (right) (n = 3).
GSEA, gene set enrichment analysis
*P < 0.05, **P < 0.01, ***P < 0.001 (Ordinary one-way ANOVA - Sidak's test).

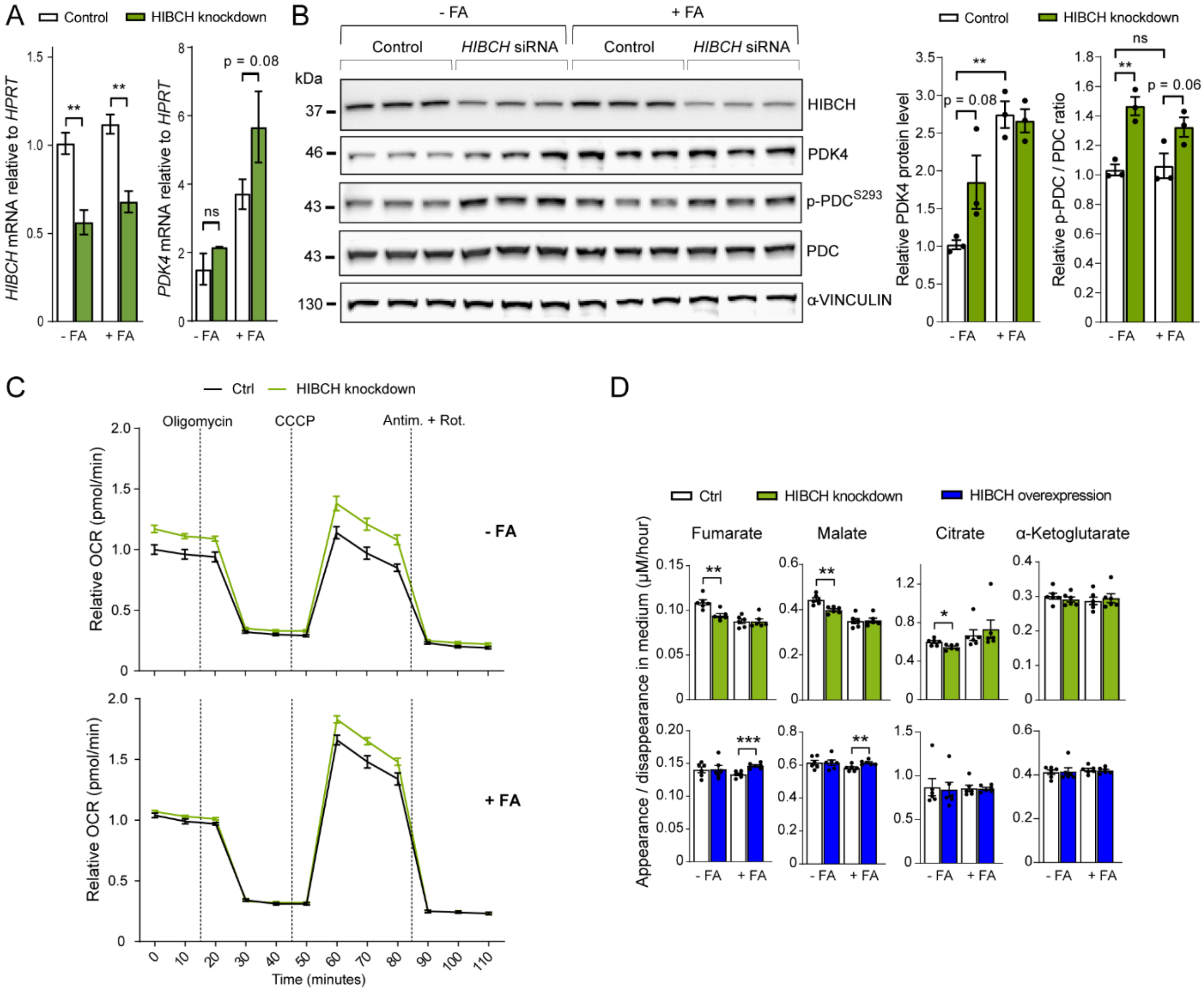

Supplementary Figure 8. HIBCH knockdown upregulates PDK4 protein expression and mitochondrial respiration and TCA cycle metabolites in cultured human hepatocytes. HepG2 (A) or Huh7 (B-D) liver cells were transfected with siRNA-mediated knockdown of HIBCH and siRNA non-targeting control or with pCMV6-HIBCH or control (pCMV6-empty vector) plasmid (0.2 µg per well in a 24-well plate) diluted in Opti-MEM® Reduced Serum Media and TransIT-X2® transfection reagent (Mirus). The cells were treated with and without free FAs (1:1 molar ratio of 50 µM PA and 50 µM OA) for 24h, before analyses. A: *HIBCH* and *PDK4* mRNA expression in HepG2 cells measured by qPCR, calculated relative to the reference gene *HPRT* (n = 3). B: Western blot showing protein levels of HIBCH, PDK4, p-PDC, PDC and α-VINCULIN (loading control) (left) and the quantitative values of HIBCH and PDK4 relative to α-vinculin and values of p-PDC/PDC ratio (right) in Huh7 cells (n = 3). C: Seahorse Cell Mito Stress Assay (OCR measurements) was performed using the Seahorse XFe96

Analyzer to assess the mitochondrial respiration in Huh7 cells (n = 10-12) Basal levels (the three first OCR measurements) were obtained, before adding oligomycin, CCCP and rotenone/antimycin A, as indicated at the top in the upper left figure. Basal respiration, ATP production, maximal respiration, spare capacity and uncoupling were calculated for each well based on the OCR measurements. D: Average net medium appearance per hour of the TCA cycle metabolites during a 24h period in Huh7 cells (n = 6).

Antim. + Rot., antimycin + rotenone; CCCP, carbonyl cyanide 3-chlorophenol hydrazone; FA, fatty acid; KD, knockdown; OCR, oxygen consumption rate.

*P < 0.05, **P < 0.01, ***P < 0.001 (unpaired t-test)

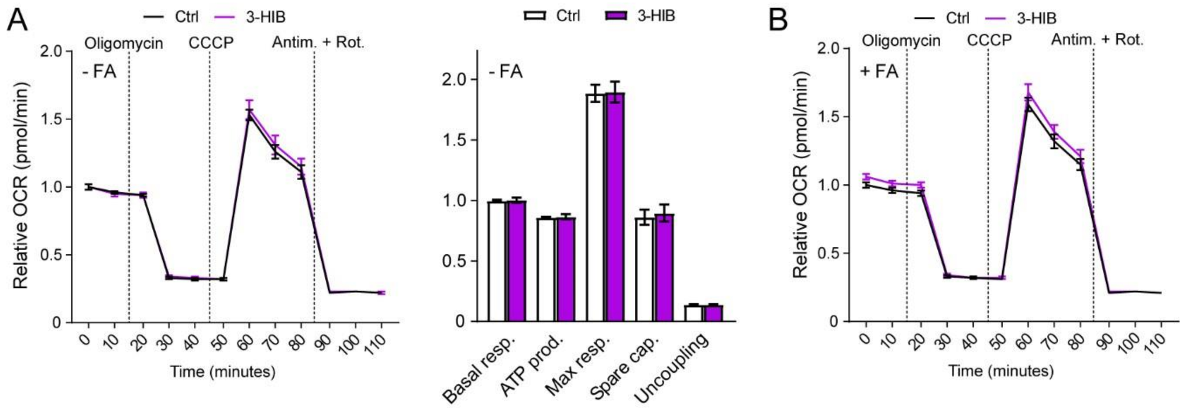

Supplementary Figure 9. Mitochondrial respiration following 3-HIB supplementation in cultured human hepatocytes. Seahorse Cell Mito Stress Assay (OCR measurements) was performed using the Seahorse XFe96 Analyzer to assess the mitochondrial respiration in Huh7 (n = 10-12) 24h after 3-HIB supplementation (final concentration 25 µM) without (A) and with (B) FA treatment. Basal levels (the three first OCR measurements) were obtained, before adding oligomycin, CCCP and rotenone/antimycin A, as indicated at the top in the upper left figure. Basal respiration, ATP production, maximal respiration, spare capacity and uncoupling were calculated for each well based on the OCR measurements (unpaired t-test test).

Antim. + Rot., antimycin + rotenone; ATP prod., adenosine triphosphate production; CCCP, carbonyl cyanide 3-chlorophenol hydrazone; FA, fatty acid treatment; resp., respiration; OCR, oxygen consumption rate; Spare cap., spare capacity.

Supplementary Tables

Supplementary Table 1. Clinical characteristics of participants in the studied cohorts.

	**CARBFUNC**	**Liver cohort**
Males/females	91/101	37/29
Age (years)	41.6 ± 8.8 (21-56)	64.6 ± 12.7 (31-90)
BMI (kg/m^2)	36.7 ± 4.8 (26.2-54.1)	32.2 ± 6.6 (22.7-45.6)
Glucose (mmol/L)	5.3 ± 0.68 (4.1-9.4)	5.7 ± 0.82 (4.2-8.4)
Insulin (nmol/L)	0.91 ± 0.34 (0.35-2.24) *	0.17 ± 0.14 (0.0038-0.628) [#]
HOMA2-IR	2.05 ± 0.81 (0.77-5.41)	NA
GIR (μmol/kg/min)	NA	58.0 ± 31.0 (11-117)
TAG (mmol/L)	1.50 ± 0.71 (0.44-4.49)	1.87 ± 0.78 (0.25-3.69)
HDL-C (mmol/L)	1.18 ± 0.25 (0.70-1.90)	1.33 ± 0.27 (0.84-2.44)
TAG/HDL-C ratio	1.39 ± 0.89 (0.34-5.61)	1.48 ± 0.70 (0.12-3.23)
HbA1c (mmol/mol)	34.48 ± 3.74 (27-55)	39.20 ± 3.41 (31.1-54.1)
% with T2D/IGT/IR	0/NA/NA	39.4/0/0
% with NAFLD/NASH	NA	33.3/36.4

Data presented as average ± SD (ranges). BMI, body mass index; GIR, glucose infusion rate (from euglycemic hyperinsulinemic clamp); IGT, impaired glucose tolerance; IR; insulin resistance; T2D, type 2 diabetes.
* Fasting C-peptide
[#] Fasting plasma insulin

Supplementary Table 2. Spearman correlations (adjusted for age and sex) for plasma metabolites and liver fat or body-mass index (BMI) in the CARBFUNC cohort.

	**Liver fat (liver/spleen density)**		**BMI (kg/m^2)**	
	Spearman's rho	p-value	Spearman's rho	p-value
**MMA**	-0.016	0.833	0.149	0.043
**Cysteine**	0.263	<0.001	0.258	<0.001
**Methionine**	0.143	0.052	0.030	0.681
**Serine**	-0.172	0.019	-0.085	0.250
**Glycine**	-0.339	<0.001	-0.167	0.023
**Tryptophan**	0.290	<0.001	0.018	0.802
**Alanine**	0.305	<0.001	0.122	0.097
**Isoleucine**	0.270	<0.001	0.097	0.190
**Leucine**	0.243	<0.001	0.040	0.584
**Valine**	0.283	<0.001	0.076	0.300
**Threonine**	0.065	0.375	0.056	0.446
**α-KG**	0.449	<0.001	0.312	<0.001
**3-HIB**	0.368	<0.001	0.024	0.750
**α-HB**	0.290	<0.001	0.079	0.281
**β-HB**	0.142	0.054	0.102	0.168
**Acetoacetate**	0.201	0.006	0.168	0.022
**BAIBA**	-0.026	0.720	-0.097	0.190

Supplementary Table 3. All reactions affected by HIBCH knockdown in constraint-based metabolic modeling of human liver cells (iHepatocytes2322).

Reaction ID	Reaction	Enzymes	Cell role	Reaction category	ModRxnId	Change KD (%)	Before KD	After 50% KD
HMR_4797	2-methyl-3-oxopropanoate [c] + H_2O [c] + NAD^+ [c] ⇒ 2 H^+ [c] + NADH [c] + methylmalonate [c]	ALDH7A1, ALDH9A1, ALDH3A2, ALDH1B1, ALDH2	produce	Valine, leucine, and isoleucine metabolism	1082	-50%	1000	500
HMR_3795	2-methyl-3-oxopropanoate [m] + CoA [m] + NAD^+ [m] ⇒ CO_2 [m] + NADH [m] + propanoyl-CoA [m]	ALDH6A1	consume	Valine, leucine, and isoleucine metabolism	807	-50%	1000	500
HMR_9046	valine [s] ⇔		consume/ produce	Exchange/demand reactions	7514	-43%	1000	574
HMR_3790	2-methyl-3-oxopropanoate [c] + H_2O [c] + O_2 [c] ⇒ H^+ [c] + H_2O_2 [c] + methylmalonate [c]	AOX1	produce	Valine, leucine, and isoleucine metabolism	801	-50%	1000	500
HMR_3789	methylmalonate [c] ⇔ methylmalonate [m]		consume/ produce	Transport reactions	6574	-50%	1000	500
HMR_3761	2-methyl-3-oxopropanoate [m] + CoA [m] + NAD^+ [m] ⇒ (R)-methylmalonyl-CoA [m] + H^+ [m] + NADH [m]	ALDH6A1	consume	Valine, leucine, and isoleucine metabolism	1090	-50%	1000	500
HMR_3748	3-methyl-2-oxobutyrate [m] + CoA [m] + NAD^+ [m] ⇒ CO_2 [m] + NADH [m] + isobutyryl-CoA [m]	BCKDHA, BCKDHB, TMEM91	consume	Valine, leucine, and isoleucine metabolism	6576	-50%	1000	500
HMR_3759	2-methyl-3-oxopropanoate [m] + H_2O [m] + NAD^+ [m] ⇒ 2 H^+ [m] + NADH [m] + methylmalonate [m]	ALDH7A1, ALDH3A2, ALDH9A1, ALDH1B1, AOX1, ALDH2	consume	Valine, leucine, and isoleucine metabolism	802	-50%	1000	500

HMR_3757	3-hydroxyisobutyrate [m] + NAD^+ [m] ⇒ 2-methyl-3oxopropanoate [m] + H^+ [m] + NADH [m]	HADH, HSD17B10, HIBADH	consume	Valine, leucine, and isoleucine metabolism	1079	-50%	1000	500

HMR_3753	H_2O [m] + methacrylyl-CoA [m] ⇒ 3hydroxyisobutyryl-CoA [m]	ECHS1, HADHA, EHHADH	consume	Valine, leucine, and isoleucine metabolism	1080	-50%	1000	500
HMR_3755	3-hydroxyisobutyryl-CoA [m] + H_2O [m] ⇒ 3hydroxyisobutyrate [m] + CoA [m] + H^+ [m]	HIBCH	produce	Valine, leucine, and isoleucine metabolism	809	-50%	1000	500
HMR_3747	AKG [c] + valine [c] ⇒ 3-methyl-2-oxobutyrate [c] + glutamate [c]	BCAT1	produce	Valine, leucine, and isoleucine metabolism	1094	-50%	1000	500
HMR_3746	3-methyl-2-oxobutyrate [c] ⇔ 3-methyl-2oxobutyrate [m]		consume/ produce	Transport reactions	1081	-50%	1000	500
HMR_6417	CoA [m] + S-(2-methylpropanoyl)-dihydrolipoamide [m] ⇔ dihydrolipoamide [m] + isobutyryl-CoA [m]	DBT	consume/ produce	Valine, leucine, and isoleucine metabolism	803	-50%	1000	500
HMR_6416	3-methyl-2-oxobutyrate [m] + H^+ [m] + lipoamide [m] ⇒ CO_2 [m] + S-(2-methylpropanoyl)-dihydrolipoamide [m]	BCKDHA, TMEM91, BCKDHB	produce	Valine, leucine, and isoleucine metabolism	808	-50%	1000	500
HMR_3744	AKG [m] + valine [m] ⇒ 3-methyl-2-oxobutyrate [m] + glutamate [m]	BCAT2	produce	Valine, leucine, and isoleucine metabolism	800	-50%	1000	500
HMR_5118	H^+ [i] + valine [c] ⇒ H^+ [m] + valine [m]		consume	Transport reactions	6662	-50%	1000	500
HMR_3763	CoA [m] + H^+ [m] + methylmalonate [m] ⇒ (R)methylmalonyl-CoA [m] + H_2O [m]	ALDH6A1	consume	Valine, leucine, and isoleucine metabolism	1077	-50%	1000	500

HMR_4497	NAD^+ [m] + hydracrylate [m] ⇒ 3-oxopropanoate [m] + H^+ [m] + NADH [m]	DHCR24	produce	Propanoate metabolism	176	-50%	1000	500
HMR_4741	3-hydroxypropionyl-CoA [m] + H_2O [m] ⇒ CoA [m] + H^+ [m] + hydracrylate [m]	HIBCH	consume	Propanoate metabolism	177	-50%	1000	500

HMR_8088	2-methyl-3-oxopropanoate [m] + glutamate [m] ⇔ AKG [m] + L-3-amino-isobutanoate [m]	ABAT	consume/ produce	Valine, leucine, and isoleucine metabolism	819	-50%	1000	500
HMR_4330	3-oxopropanoate [m] + glutamate [m] ⇔ AKG [m] + beta-alanine [m]	ABAT	consume/ produce	Beta-alanine metabolism	1046	-50%	1000	500
HMR_8418	2,6-dimethylheptanoyl-carnitine [c] + L-carnitine [m] ⇔ 2,6-dimethylheptanoyl-carnitine [m] + L-carnitine [c]		consume/ produce	Carnitine shuttle (mitochondrial)	2389	-50%	1000	500
HMR_3527	2(S),6-dimethyl-heptanoyl-CoA [m] + FAD [m] ⇒ 2,6dimethyl-trans-2-heptenoyl-CoA [m] + $FADH_2$ [m]	ACADM, ACADL	consume	Beta oxidation of branched-chain fatty acids (mitochondrial)	2868	-50%	1000	500
HMR_3528	2,6-dimethyl-trans-2-heptenoyl-CoA [m] + H_2O [m] ⇒ 3(S)-hydroxy-2(S),6-dimethyl-heptanoyl-CoA [m]	EHHADH, ECHS1, HADHA	consume	Beta oxidation of branched-chain fatty acids (mitochondrial)	2869	-50%	1000	500
HMR_3529	3(S)-hydroxy-2(S),6-dimethyl-heptanoyl-CoA [m] + NAD^+ [m] ⇒ 3-oxo-(2S)-methylisocapryloyl-CoA [m] + H^+ [m] + NADH [m]	HADHA, HADH, HSD17B10	consume	Beta oxidation of branched-chain fatty acids (mitochondrial)	2870	-50%	1000	500
HMR_3530	3-oxo-(2S)-methylisocapryloyl-CoA [m] + CoA [m] ⇒ 4methyl-pentanoyl-CoA [m] + propanoyl-CoA [m]	ACAA2, HADHB, HADHA	consume	Beta oxidation of branched-chain fatty acids (mitochondrial)	2871	-50%	1000	500

HMR_3531	4-methyl-pentanoyl-CoA [m] + FAD [m] ⇒ 4-methyltrans-2-pentenoyl-CoA [m] + $FADH_2$ [m]	ACADL, ACADM	consume	Beta oxidation of branched-chain fatty acids (mitochondrial)	2872	-50%	1000	500
HMR_3532	4-methyl-trans-2-pentenoyl-CoA [m] + H_2O [m] ⇒ 3(S)-hydroxy-4-methyl-pentanoyl-CoA [m]	EHHADH, HADHA, ECHS1	consume	Beta oxidation of branched-chain fatty acids (mitochondrial)	2873	-50%	1000	500
HMR_3533	3(S)-hydroxy-4-methyl-pentanoyl-CoA [m] + NAD^+ [m] ⇒ 3-oxo-4-methyl-pentanoyl-CoA [m] + H^+ [m] + NADH [m]	HADH, HSD17B10, HADHA	consume	Beta oxidation of branched-chain fatty acids (mitochondrial)	2874	-50%	1000	500
HMR_3534	3-oxo-4-methyl-pentanoyl-CoA [m] + CoA [m] ⇒ acetyl-CoA [m] + isobutyryl-CoA [m]	HADHA, ACAA2, HADHB	consume	Beta oxidation of branched-chain fatty acids (mitochondrial)	2875	-50%	1000	500
HMR_8089	L-3-amino-isobutanoate [m] ⇒ L-3-aminoisobutanoate [c]	ABAT	consume	Transport reactions	6817	-50%	1000	500
HMR_8090	L-3-amino-isobutanoate [c] ⇒ L-3-aminoisobutanoate [s]		produce	Transport reactions	6299	-50%	1000	500
HMR_9307	2,6-dimethylheptanoyl-carnitine [s] ⇔		consume/ produce	Exchange/demand reactions	7732	-50%	1000	500
